# Microbiota Supplementation with *Bifidobacterium* and *Lactobacillus* Modifies the Preterm Infant Gut Microbiota and Metabolome: An Observational Study

**DOI:** 10.1016/j.xcrm.2020.100077

**Published:** 2020-08-25

**Authors:** Cristina Alcon-Giner, Matthew J. Dalby, Shabhonam Caim, Jennifer Ketskemety, Alex Shaw, Kathleen Sim, Melissa A.E. Lawson, Raymond Kiu, Charlotte Leclaire, Lisa Chalklen, Magdalena Kujawska, Suparna Mitra, Fahmina Fardus-Reid, Gustav Belteki, Katherine McColl, Jonathan R. Swann, J. Simon Kroll, Paul Clarke, Lindsay J. Hall

**Affiliations:** 1 Gut Microbes & Health, Quadram Institute Bioscience, Norwich Research Park, Norwich, UK; 2 Department of Medicine, Section of Pediatrics, Imperial College London, London, UK; 3 Leeds Institute of Medical Research, University of Leeds, Leeds, UK; 4 Department of Surgery and Cancer, Faculty of Medicine, Imperial College London, London, UK; 5 Neonatal Intensive Care Unit, The Rosie Hospital, Cambridge University Hospitals NHS Foundation Trust, Cambridge, UK; 6 Neonatal Intensive Care Unit, Norfolk and Norwich University Hospital, Norwich, UK; 7 Norwich Medical School, University of East Anglia, Norwich, UK; 8 Chair of Intestinal Microbiome, School of Life Sciences, Technical University of Munich, Freising, Germany; 9 ZIEL – Institute for Food & Health, Technical University of Munich, Freising, Germany

**Keywords:** preterm infant, *Bifidobacterium*, *Lactobacillus*, probiotic, supplementation, human milk oligosaccharides, pH, microbiota, metabolites, pathobionts

## Abstract

Supplementation with members of the early-life microbiota as “probiotics” is increasingly used in attempts to beneficially manipulate the preterm infant gut microbiota. We performed a large observational longitudinal study comprising two preterm groups: 101 infants orally supplemented with *Bifidobacterium* and *Lactobacillus* (Bif/Lacto) and 133 infants non-supplemented (control) matched by age, sex, and delivery method. 16S rRNA gene profiling on fecal samples (n = 592) showed a predominance of *Bifidobacterium* and a lower abundance of pathobionts in the Bif/Lacto group. Metabolomic analysis showed higher fecal acetate and lactate and a lower fecal pH in the Bif/Lacto group compared to the control group. Fecal acetate positively correlated with relative abundance of *Bifidobacterium,* consistent with the ability of the supplemented *Bifidobacterium* strain to metabolize human milk oligosaccharides into acetate. This study demonstrates that microbiota supplementation is associated with a *Bifidobacterium*-dominated preterm microbiota and gastrointestinal environment more closely resembling that of full-term infants.

## Highlights

*Bifidobacterium* dominates the gut microbiota in supplemented preterm infants
Supplemented preterm infants have lower abundance of potential pathobionts
Metabolomic analysis show higher fecal acetate and lower pH in supplemented infants
*In vitro* and genomic analysis confirm HMO metabolism in *Bifidobacterium* supplement

## Graphical Abstract

**Figure:**
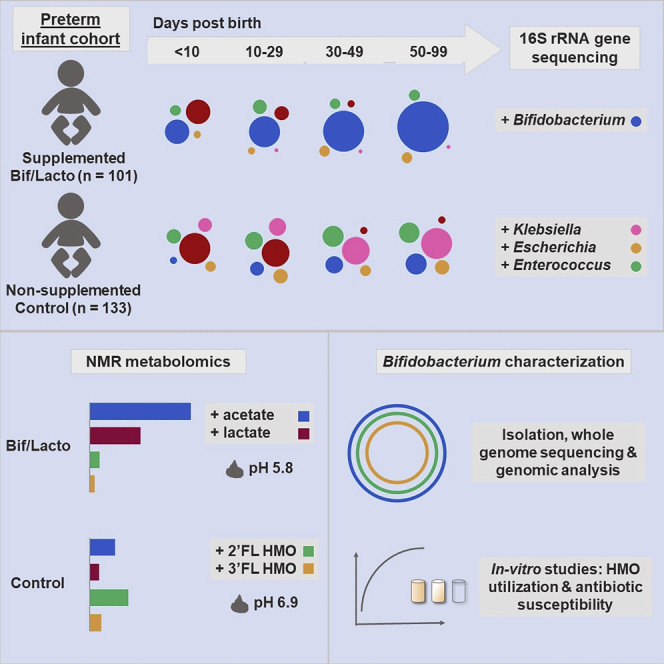

## Teaser

Alcon-Giner et al. show that probiotic supplementation in preterm infants leads to a *Bifidobacterium*-dominated gut microbiota, elevated acetate and lactate levels, and lower fecal pH, with concurrent reduction in pathobionts. *Bifidobacterium* isolation confirms strain presence; genomic and *in vitro* studies indicate strain encoded enzymatic clusters allowing growth on HMOs.

## Introduction

Infants born <37 weeks gestation are defined as preterm and account for 1 in 9 births globally.^1^ Compared to full-term infants, preterm infants are more often born via Caesarean section, have an underdeveloped immune system, receive numerous courses of antibiotics, and reside in neonatal intensive care units (NICUs), all of which disrupt the establishment of the early-life gut microbiota.^2^, ^3^, ^4^ This altered gut microbial ecosystem has been linked to an increased risk of serious morbidity during the NICU stay, including necrotizing enterocolitis (NEC),^5^ late-onset sepsis (LOS),^6^ and later-life health problems such as asthma and eczema.^7^^,^^8^

Abnormal patterns of bacterial colonization are common in the preterm infant gut, which is dominated by genera containing potentially pathogenic bacteria (i.e., pathobionts) such as *Staphylococcus*, *Klebsiella*, *Escherichia*, and *Clostridium*.^2^^,^^9^ These infants are also characterized by a low abundance or absence of the beneficial genera *Bifidobacterium*, which is dominant in the full-term infant gut.^10^^,^^11^ Thus, interventions to “normalize” the preterm gut microbiota are an attractive proposition to improve health and prevent disease in preterm infants.

Oral administration of commensal infant bacteria via probiotic^12^ supplementation is one approach to encourage gut colonization by beneficial members of the early life microbiota. Systematic review and meta-analysis of randomized controlled trials and observational studies have reported that probiotic supplementation reduces NEC, sepsis, and all-cause mortality in preterm infants.^13^^,^^14^ However, one of the largest trials carried out in the UK found no evidence of benefit.^15^ Despite the positive outcome obtained in previous systematic reviews and meta-analyses, a 2018 survey of all 58 UK tertiary-level NICUs found only 10 NICUs (17%) were routinely using probiotics.^16^ While clinical studies have demonstrated the potential of probiotics to reduce NEC incidence, there are few that have also performed accompanying longitudinal microbiota profiling (often with relatively low numbers of infants^17^, ^18^, ^19^) to determine the impact of supplementation on gut microbiota composition, and little or none that have examined the corresponding metabolome of preterm infants^20^ nor included whole-genome sequencing of probiotic bacteria isolated from the supplement used or from the recipient infants.

A recent clinical audit at the Norfolk and Norwich University Hospital NICU found rates of NEC fell from 7.5% to 3.1%, and rates of LOS fell from 22.6% to 11.5% when comparing the 5 years before and 5 years after the initiation of routine probiotic use with a combined *Bifidobacterium* and *Lactobacillus* supplement.^21^ Building on these important clinical observations, we aimed to explore the gut microbiota composition and fecal metabolome in these preterm infants receiving routine probiotic supplementation compared to preterm infants from NICUs not using probiotic supplementation.

Thus, we carried out an observational study comparing longitudinal samples from two cohorts of preterm infants; 101 orally supplemented with a combination of *Bifidobacterium* and *Lactobacillus* (Infloran, given twice daily with the first enteral feed) at the Norfolk and Norwich University Hospital NICU and 133 non-supplemented infants from NICUs not using probiotic supplementation. Cohorts were approximately matched by gestational age, sex, delivery method, and sample collection time across the four tertiary-level NICUs. 16S rRNA gene profiling was used to determine the fecal bacterial composition (n = 592), and paired ^1^H nuclear magnetic resonance (NMR) spectroscopy was used to measure the metabolic content of the fecal samples; this included metabolites of microbial, host, and maternal origin (n = 157). To further evaluate the supplemented strains, we performed whole-genome sequencing to compare supplemented strains to isolates obtained from preterm infants, alongside *in vitro* studies to define factors that may impact supplemented strains and their persistence within the preterm microbiota.

## Results

### Study Design

**Figure 1:**
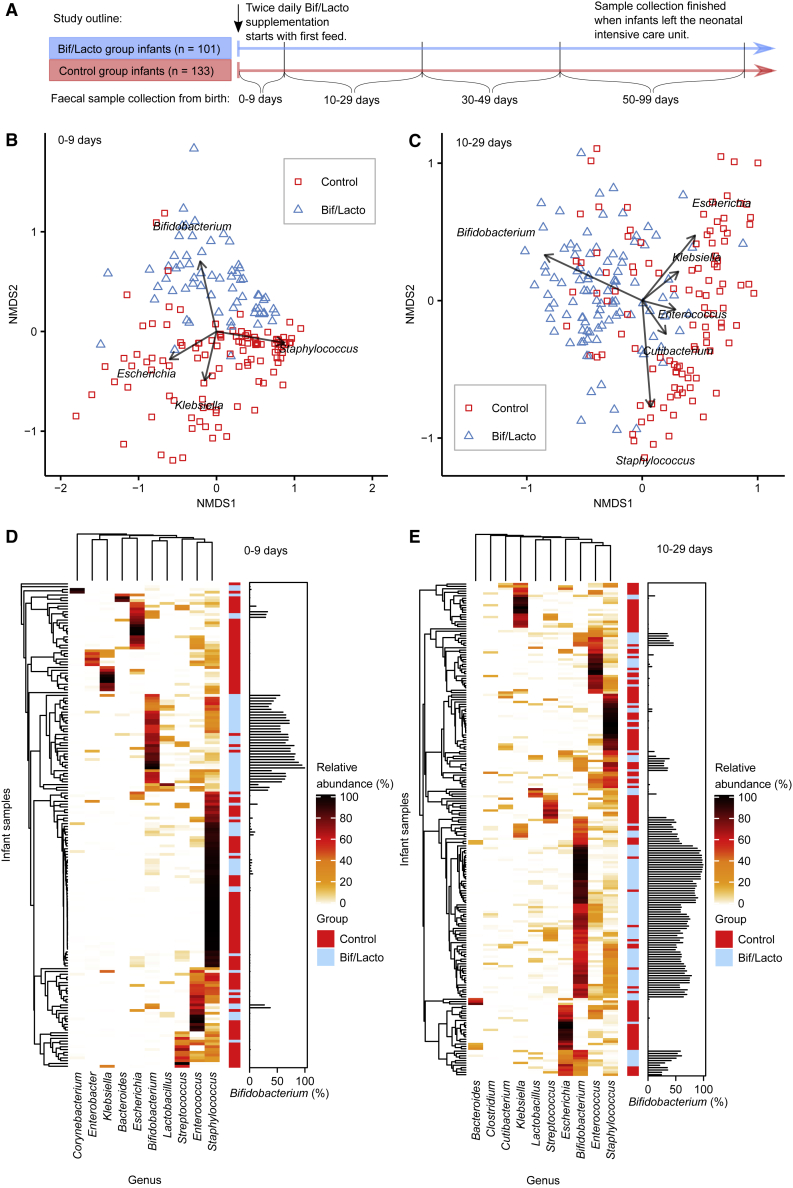
Premature Infant Gut Microbiota Clustering and Genus Composition NMDS (non-metric multidimensional scaling) analysis clustered with a Bray-Curtis dissimilarity. Arrows and genus labels on the NMDS plots indicate bacterial genera driving the separation of points on the NMDS plots. Heatmaps showing the ten genera with highest proportional abundance. Heatmap rows were clustered by total microbiota similarity using Bray-Curtis dissimilarity and the columns clustered by genera that occur more often together. Side bar plots show the proportional abundance of *Bifidobacterium* in each sample. (A) Study outline and sample collections times. Infloran supplementation was given until 34 weeks old, except for very low-birth-weight infants (<1,500 g) who received it until discharge. The control group was not given supplementation. (B) NMDS plot of infant fecal microbiota at 0–9 days (control: n = 110, Bif/Lacto: n = 64). (C) NMDS plot of infant fecal microbiota at 10–29 days (control: n = 109, Bif/Lacto: n = 100). (D) Heatmap showing infant fecal microbiota at 0–9 days (control: n = 110, Bif/Lacto: n = 64). (E) Heatmap showing infant fecal microbiota at 10–29 days (control: n = 109, Bif/Lacto: n = 100). See also Figure S1, Data S1–S3, and Tables S1 and S3.

Fecal samples were collected from NICU-resident preterm infants receiving a daily oral supplementation containing *Bifidobacterium bifidum* and *Lactobacillus acidophilus* (Bif/Lacto group), and from a group of similarly aged preterm infants (control group) from three other NICUs that did not offer supplementation. Although there are caveats associated with this observational study design, it did avoid potential cross-contamination among study groups, which has been reported previously in other probiotic studies where study groups reside within the same NICU.^15^^,^^22^, ^23^, ^24^ Samples were collected corresponding to four time points at 0–9, 10–29, 30–49, and 50–99 days of age from birth (Figure 1A).

### Supplementation with Early Life Microbiota Members Influences Preterm Gut Microbiota Composition

The preterm gut is typically dominated by pathobionts such as *Enterobacter*, *Escherichia*, and *Klebsiella*. We sought to determine whether preterm infants supplemented with *Bifidobacterium and Lactobacillus*, bacterial species associated with a healthy term infant gut, showed a modified preterm microbiota profile. Fecal bacterial composition was determined by 16S rRNA gene sequencing. Genus level clustering of samples using non-metric multidimensional scaling (NMDS) indicated clear variation in the microbiota profiles between Bif/Lacto supplemented infants and controls (Figures 1B and 1C; Figures S1A and S1B). The microbiota composition of Bif/Lacto and control samples differed significantly at each of the four time points (PERMANOVA 0–9 days: p < 0.01, R2 = 8.2%; 10–29 days: p < 0.01, R2 = 12%; 30–49 days: p < 0.01, R2 = 15%; 50–99 days: p < 0.01, R2 = 12%). The clustering of the Bif/Lacto group was driven by the genus *Bifidobacterium*, while the genera driving the clustering of the control group included *Staphylococcus*, *Escherichia*, and *Klebsiella* (Figures 1B and 1C; Figures S1A and S1B). Given that microbial succession patterns differ between extremely and moderately premature infants, we divided the dataset into infants born under or over 28 weeks gestational age. Visualized as NMDS plots, these show similar separation patterns between Bif/Lacto and control groups driven by *Bifidobacterium* at 0–9 and 10–29 days regardless of gestational age with some greater complexity visible at 10–29 days of age (Figures S3A–S3D). To test for the effects of method used to normalize the 16S sequence data, we also normalized to account for differences in sampling depth between samples using centered-log ratio transformations and variance stabilization transformation. NMDS plots of at 0–9 and 10–29 days of age using centered-log ratio transformed data (Data S2A and S2B) and at 0–9 and 10–29 days of age using variance stabilization transformed (Data S2C and S2D) show similar results to using rarefied data (Figures 1B and 1C). Relative abundance of *Bifidobacterium* also showed similar differences between groups regardless of normalization method (Data S2E and S2F).

Notably, while hospital NICUs may differ in their “environmental” microbiota in ways that may influence infant colonization, NMDS indicated no differences in the microbiota composition between infant samples from the three control hospital NICUs involved (Data S3A–S3D; Table S10). A PERMANOVA multivariate analysis including the infants from all four NICUs showed no significant influence of NICUs at 0–9 and 10–29 days after taking account of the Bif/Lacto and control study groups (Table S10). PERMANOVA multivariate analysis followed by multilevel pairwise comparison showed that the differences between NICUs was due to differences between the Bif/Lacto NICU (Norfolk and Norwich) and the three control NICUs, not between the three control NICUs (Table S11).

We also examined the ten most abundant genera by relative abundance at each time point clustered using Bray-Curtis dissimilarity (Figures 1D and 1E; Figures S1C and S1D), showing infant samples clustered into six main groups based on a single dominant bacterial genus; *Bifidobacterium*, *Escherichia*, *Enterococcus*, *Klebsiella*, *Staphylococcus*, or *Streptococcus.* These data indicate that the introduction of *Bifidobacterium* promotes changes in the composition of the preterm gut microbiota.

### Oral Bif/Lacto Supplementation Influences Bacterial Genus Abundance and Bacterial Diversity

**Figure 2:**
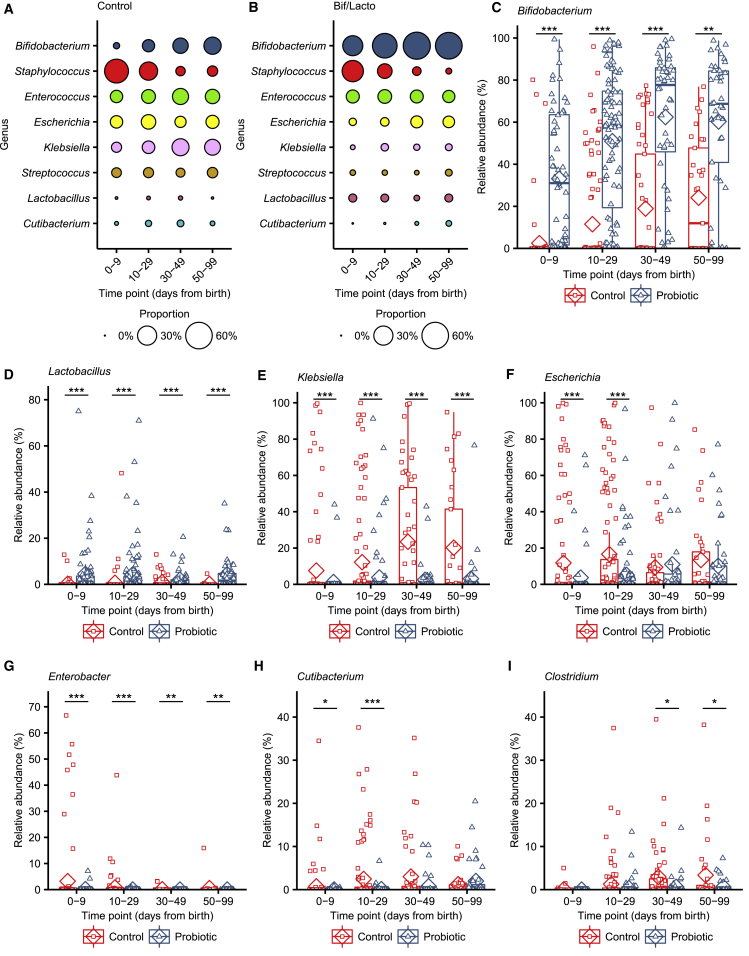
Genus Abundance between Bif/Lacto and Control Groups (A) Bubble plots show the mean group abundance of the common bacterial genera at each time point in the control group and the Bif/Lacto group. (C) Relative abundance of *Bifidobacterium*. (D) Relative abundance of *Lactobacillus*. (E) Relative abundance of *Klebsiella*, (F) Relative abundance of *Escherichia*. (G) Relative abundance of *Enterobacter* (H) Relative abundance of *Cutibacterium* (I) Relative abundance of *Clostridium*. For all plots: 0–9 days (control: n = 110, Bif/Lacto: n = 64); 10–29 days (control: n = 109, Bif/Lacto: n = 100); 30–49 days (control: n = 57, Bif/Lacto: n = 48); 50–99 days (control: n = 33, Bif/Lacto: n = 41). Boxplots show group median and interquartile range, diamonds indicate the group mean, and individual points highlight individual infant samples. Asterisks represent p values: ∗p < 0.05, ∗∗p < 0.01 ∗∗∗p < 0.001. See also Figure S2 and Data S4–S6.

We sought to further define the genus composition based on relative abundance and diversity measures underlying these changes in microbiota composition. *Bifidobacterium* dominated the microbiota of the Bif/Lacto group with high relative abundance at all time points compared to the control group (Figures 2A–2C). This indicated that the supplemented strain may persist in the preterm microbiota and/or encourage colonization of other *Bifidobacterium* spp. Surprisingly, *Lactobacillus* was only detected in a minority of infants but with a higher relative abundance in Bif/Lacto infants compared to the control group at all time points (Figure 2D), which may indicate a more transient and limited persistence for this strain. The relative abundance of bacteria such as *Klebsiella*, *Escherichia*, and *Enterobacter* was lower in Bif/Lacto infants compared to control infants at earlier time points 0–9 and 10–29 days of age (Figures 2E–2G), with *Klebsiella* still lower at 30–99 days of age (Figure 2E). *Clostridium* was also lower at 30–49 and 50–99 days of age in Bif/Lacto infants (Figure 2I). *Staphylococcus* was initially abundant in both groups but rapidly decreased as the infants aged (Figure 2A and 2B; Figure S5F).^25^ The skin-associated commensal *Cutibacterium* was also found in higher relative abundance in control infants at 0–9 and 10–29 days of age (Figure 2H). Comparing the prevalence between groups showed that the percentage of infants with detectable *Bifidobacterium* and *Lactobacillus* were higher while *Klebsiella*, *Escherichia*, *Enterococcus*, and *Clostridium* were lower in the Bif/Lacto group compared to control infants (Data S6A–S6F). This was particularly notable for *Lactobacillus*, which was highly prevalent in Bif/Lacto infants despite only a few infants having a large relative abundance of *Lactobacillus*. These data suggest that the oral supplementation impacts the microbial ecosystem patterns, displacing other potentially pathogenic bacteria more typical of the preterm gut.

Species level analysis of the 16S rRNA gene data revealed a relative abundance of *Bifidobacterium bifidum* (Figure 4A) and *Lactobacillus acidophilus* (Figure S4D) in the Bif/Lacto group. This was validated by performing bacterial isolation and whole-genome sequencing (see below and Figure 4B). The genus *Staphylococcus* matched to *S. epidermidis* and *S. haemolyticus*, bacterial residents on the skin, indicating that these originate from initial colonization of skin-associated bacteria (Figures S5G and S5H).

When examining diversity measures (Shannon and Inverse Simpson diversity), values were initially higher at 0–9 days in Bif/Lacto compared to control infants (Figures S2B and S2C), although the abundance of *Bifidobacterium* was not correlated with the number of bacterial genera detected (Figure S2D). At later time points (30–99 days of age; Figures S2B and S2C), the diversity values of the Bif/Lacto were lower than the control group, which may correlate with the increasing *Bifidobacterium* abundance (Figures S2E, S2F, S2H, and S2I). These data indicate (relative abundance) dominance of *Bifidobacterium* within the preterm microbiota results in a microbiota with low diversity.

### External Factors Including Gestational Age, Birth Weight, and Antibiotics Negatively Affect *Bifidobacterium* Abundance in Bif/Lacto Infants

Previous studies have indicated that factors, such as gestational age and antibiotics,^26^ significantly influence the developing early life gut microbiota, with preterm infants representing an infant cohort highly vulnerable to multiple microbiome-modulating factors. Comparing overall genus composition using PERMANOVA multivariate analysis (using a Bray-Curtis matrix; Table S10) indicated that oral supplementation was the most significant variable explaining variance in the infant microbiota at each time point. Delivery method contributed a small proportion of variance at 0–9 days. Birth weight significantly contributed to variance in microbiota composition at 0–9, 10–29, and 30–49 days of age (Table S10).

**Figure 3:**
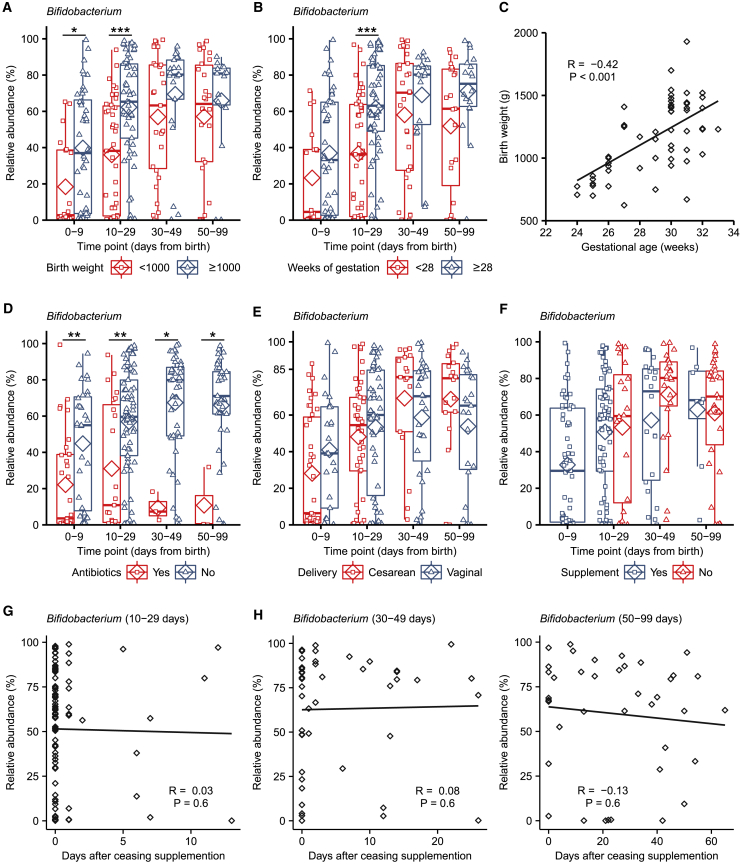
Effects of Birth Weight, Antibiotic Use, Delivery Mode, and Bifidobacterium Colonization in Bif/Lacto Group Infants (A) *Bifidobacterium* abundance between very low birth weight (<1,000 g) and low birth weight (>1,000 g) in Bif/Lacto infants (N = 0–9: <1,000 = 20, ≥1,000 = 44; 10–29: <1,000 = 43, ≥1,000 = 57; 30–49: <1,000 = 27, ≥1,000 = 21; 50–99: <1,000 = 26, ≥1,000 = 15). (B) *Bifidobacterium* abundance between very low gestational age (<28 weeks) and low gestational age (≥28 weeks) Bif/Lacto infants (N = 0–9: <1,000 = 18, ≥1,000 = 46; 10–29: <1,000 = 43, ≥1,000 = 57; 30–49: <1,000 = 29, ≥1,000 = 19; 50–99: <1,000 = 23, ≥1,000 = 18). (C) Infant birth weight in grams correlated with gestational age in weeks (n = 100). (D) *Bifidobacterium* abundance in Bif/Lacto infants receiving antibiotics at the time of sample collection (N = 0–9: Yes = 33, No = 31; 10–29: Yes = 23, No = 77; 30–49: Yes = 3, No = 44; 50–99: Yes = 3, No = 37). (E) *Bifidobacterium* abundance in Bif/Lacto infants delivered by caesarean or vaginal birth (N = 0–9: C = 39, V = 25; 10–29: C = 46, V = 54; 30–49: C = 17, V = 31; 50–99: C = 18, V = 23). (F) *Bifidobacterium* abundance in Bif/Lacto infants still receiving or no longer receiving supplementation (N = 0–9: Yes = 63; 10–29: Yes = 77, No = 20; 30–49: Yes = 22, No = 23; 50–99: Yes = 8, No = 30). (G) *Bifidobacterium* abundance in Bif/Lacto infants by days after ceasing supplementation at 10–29 days of age (n = 97). (H) *Bifidobacterium* abundance in Bif/Lacto infants by days after ceasing supplementation at 30–49 days of age (n = 45). (I) *Bifidobacterium* abundance in Bif/Lacto infants by days after ceasing supplementation at 50–99 days of age (n = 38). Boxplots show group median and interquartile range, diamonds indicate the group mean, and individual points highlight individual infant samples. Asterisks represent p values: ∗p < 0.05, ∗∗p < 0.01 ∗∗∗p < 0.001. See also Figure S3 and Data S7.

Focusing on *Bifidobacterium* as the dominant bacteria in the Bif/Lacto group, we noted that infants with a birth weight ≥1,000 g showed higher relative abundance of *Bifidobacterium* at 0–29 days (Figure 3A; Figure S3A). This was also the case at 10–29 days of age in Bif/Lacto infants born at a gestational age ≥28 weeks (Figure 3B), indicating that the underdeveloped preterm gut may not represent an optimal niche for *Bifidobacterium* persistence. Birth weight and gestational age were closely correlated (Figure 3C) and correlated inversely with length of NICU stay (Figures S3E and S3F) as smaller infants remained in NICU for a longer time. Higher *Bifidobacterium* proportions in control infants with birth weights ≥1,000 g compared to those of <1,000 g supports this hypothesis (Figure S3A). There was no difference in length of stay in NICU between Bif/Lacto and control infants (Table S1; Data S4E).

Preterm infants receive numerous antibiotics over the course of their NICU stay. Within the Bif/Lacto infants *Bifidobacterium* abundance was lower in infants currently being treated with antibiotics at all time points compared to those not receiving antibiotics, indicating antibiotic susceptibility of this genus (Figure 3D). In contrast, the relative abundance of *Staphylococcus*, *Klebsiella*, and *Escherichia* was unchanged in infants receiving antibiotics, suggesting these were resistant to antibiotic treatment (Figures S3I–S3K).

Emergency Caesarean sections for maternal or fetal indications account for a large number of preterm births, and previous studies have indicated that Caesarean-section delivery can directly interrupt the transfer of maternal microbes (e.g., *Bifidobacterium*) to infants.^10^^,^^27^ We observed no significant difference in the relative abundance of *Bifidobacterium* within the Bif/Lacto group in infants born by vaginal or cesarean birth (Figure 3E). Gestational age, current antibiotic treatment, and delivery method did not significantly alter *Bifidobacterium* proportions in control infants; however, the low abundance of this bacteria in this cohort make robust statistical analysis difficult (Figures S3B–S3D).

In Bif/Lacto infants, supplementation ceased when infants reached a post-conceptual age of 34 weeks. However, no reduction was observed in the relative abundance of *Bifidobacterium* in samples collected from these infants after oral supplementation had ceased (Figure 3F), with proportions maintained for up to 60 days (Figures 3G–3I). *Bifidobacterium* species and strain level analysis using bacterial isolation and whole-genome sequencing is examined in more detail in the following section to assess the potential persistence of the supplemented strain in these infants.

Diet is proposed to be one of the major factors modulating the early life microbiota, with significant differences between formula and breast-fed infants.^28^ Unusually, almost all infants recruited to this study were fed either their own mothers’ breast milk (BM), their mothers’ BM and donor BM (DBM) in combination, or BM supplemented with preterm cows’ milk-based formula. However, there were group differences in the prevalence of exclusive feeding of mother’s BM and duration of antibiotic treatment between the Bif/Lacto and control groups of infants (Table S1). Seventy percent of the Bif/Lacto group infants received an exclusive BM-based diet (70%), while the majority of infants in the control group received a mixed BM and DBM (BM+DBM) diet (51%) or an exclusively BM diet (27%) (Table S1). These differences may act as a confounder between the study cohorts, with the pasteurization process of DBM impacting the milk microbiome.^29^ However, in this study use of DBM was always given to supplement shortfalls in mothers’ own BM, with the infant still receiving BM. Indeed, we observed no differences in the overall microbiota composition in either Bif/Lacto infants or control infants between those fed mothers’ BM compared to those fed a combination of mothers’ BM and DBM (Data S4A–S4D). PERMANOVA multivariate analysis indicated that type of infant diet fed at time of sample collection did not contribute to the differences in overall microbiota composition between Bif/Lacto and control groups (Table S10). To further investigate the potential confounding effect of diet, a sensitivity analysis restricted to infants only receiving mother’s BM at the time of sample collection showed similar differences in relative abundance of *Bifidobacterium*, *Lactobacillus*, *Klebsiella*, *Escherichia*, *Enterococcus*, and *Clostridium* between Bif/Lacto and control infants as seen in all infants (Data S5G–S5L). Additionally, PERMANOVA indicated no consistent effects of diet on the relative abundance of *Bifidobacterium*, and none of the relative abundance of potential pathobionts *Klebsiella* and *Escherichia* between Bif/Lacto and control group infants (Table S12). Regarding exclusively formula-fed infants, only a very small number of infants were recruited in this study (i.e., 7 out of 234), which may explain our findings that the relative abundance of *Bifidobacterium* was not consistently affected by diet (Table S12). There were no differences between infants fed mother’s BM or mixed mother’s BM and DBM.

Investigating the potential confounding effects of duration of antibiotic treatment using PERMANOVA multivariate analysis showed that antibiotic treatment duration did not contribute to the differences in overall microbiota composition between Bif/Lacto and control groups (Table S10). A sensitivity analysis restricted only to infants receiving short duration antibiotic treatment showed similar differences in relative abundance of *Bifidobacterium*, *Lactobacillus*, *Klebsiella*, *Escherichia*, *Enterococcus*, and *Clostridium* between Bif/Lacto and control infants as seen in all infants (Data S5A–S5F). Additionally, testing the effects of group, diet, and antibiotic duration on the relative abundance of either *Bifidobacterium*, *Klebsiella*, or *Escherichia* using PERMANOVA (Table S12) indicated a small influence on *Bifidobacterium* abundance due to antibiotic duration at 10–29, 30–49, and 50–99 days of age, while *Klebsiella* and *Escherichia* abundance was unaffected by antibiotic duration.

### Bif/Lacto Infants Show Persistence of the *Bifidobacterium bifidum* Infloran Strain, Which Correlated with Human Breast Milk Metabolism and Routine Supplementation

**Figure 4:**
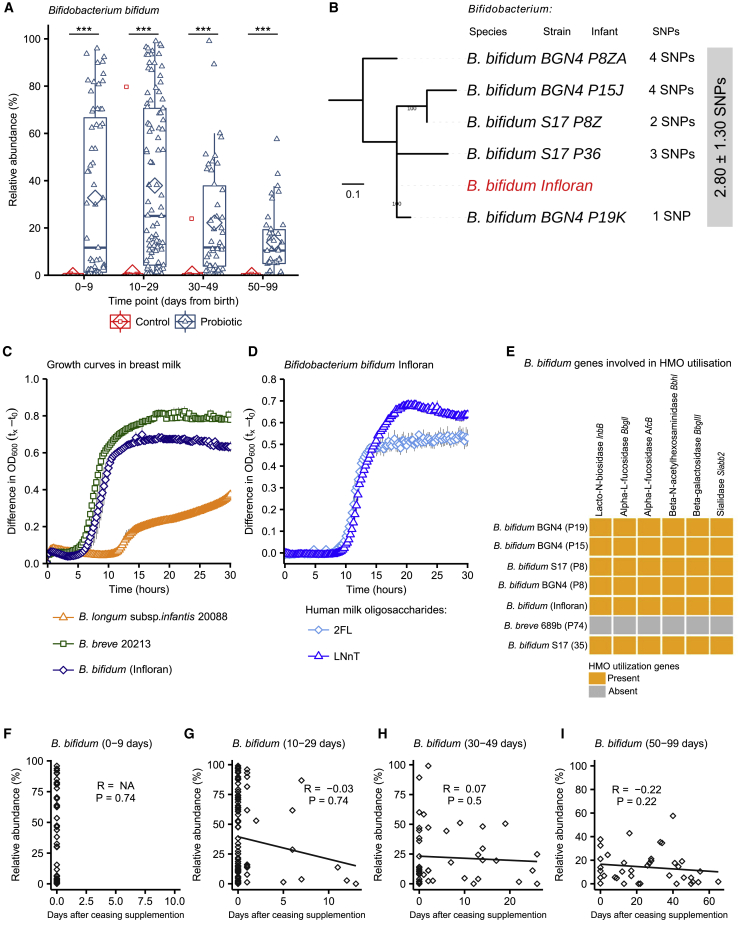
Comparison of *B. bifidum* Genomes and Phenotypic Characterization of *B. bifidum* Infloran Strain (A) *B. bifidum* abundance in Bif/Lacto and control group infants (0–9 days (control: n = 62, Bif/Lacto: n = 63); 10–29 days (control: n = 70, Bif/Lacto: n = 97); 30–49 days (control: n = 38, Bif/Lacto: n = 46); 50–99 days (control: n = 22, Bif/Lacto: n = 39)). (B) Mid-point rooted maximum-likelihood tree based on 12 SNPs called via reference-based approach (strain Infloran as the reference genome) from 5 *B. bifidum* genomes. The gray box denotes pairwise SNP distance between these 6 genomes. Data: mean ± SD. (C) Growth curves of *B. bifidum* Infloran, *B. breve* 20213, and *B. longum* subsp. *infantis* 20088, in whole human milk. (D) Growth curves *B. bifidum* Infloran in human milk oligosaccharides (HMO) Lacto-N-tetraose and 2-fucosyllactose. (E) Heatmap representing *B. bifidum* genes involved in utilization of human milk oligosaccharides. (F–I) Correlation between *B. bifidum* abundance and days after ceasing receiving supplementation (0–9 days: n = 63; 10–29 days: n = 97; 30–49 days: n = 46; 50–99 days: n = 39). Boxplots show group median and interquartile range, diamonds indicate the group mean, and individual points highlight individual infant samples. Asterisks represent p values: ∗∗∗p < 0.001. See also Figures S4 and S5 and Tables S2 and S5.

Our data so far indicated a dominance of *Bifidobacterium* in the Bif/Lacto cohort and lower relative abundance of *Lactobacillus* in the preterm gut. To probe this with greater resolution, we compared the abundance of species present within these two genera. *B. bifidum* was highly abundant in Bif/Lacto infants, while only being abundant in 2/133 control infants (Figure 4A). *Bifidobacterium breve* was also more abundant in the Bif/Lacto supplemented group (Figure S4A), with *Bifidobacterium longum* present in a small number of infants from both groups (Figure S4A). *B. bifidum* relative abundance declined with increasing infant age (Figure 4A), with concurrent increases in *B. breve* (Figure S4A). As *B. breve* coexisted with, rather than replaced (Figure S4I) *B. bifidum* this suggests close species interactions, potentially via metabolite cross-feeding. In contrast, while the relative abundance of *Lactobacillus acidophilus* Infloran strain was significantly enhanced in Bif/Lacto infants (Figure S4D), abundance decreased to zero within days after cessation of supplementation (Figures S4E–S4H), indicating low-level persistence of this species. To confirm species classification of *Lactobacillus acidophilus Infloran* strain, genome comparison analysis was performed against other *Lactobacillus* species (Figures S10D and S10E).

Previous research studies have shown that colonization of the gut by probiotic bacteria may vary depending on the strains used, mode of administration, dose, and inclusion of prebiotics.^30^ To understand whether the *B. bifidum* Infloran strain was able to persist within the preterm microbiota after supplementation, we obtained nine *Bifidobacterium* isolates cultured from fecal samples from seven Bif/Lacto infants (five of them received supplementation at the time of sample collection and two had stopped supplementation). We performed whole-genome sequencing on all the isolates and compared their sequence to the *B. bifidum* Infloran strain. Core-genome single nucleotide polymorphism (SNP) analysis indicated the five *B. bifidum* isolates were identical at 0 SNP difference (based on 87 core genes; Figure S5A). Reference-based genome mapping of whole-genome sequences of five *B. bifidum* genomes to *B. bifidum* Infloran strain (as reference genome; Figure 4B) indicated a near-identical similarity (mean SNP distance: 2.80 ± 1.30 SNPs), strongly suggesting they belong to the same bacterial strain (i.e., Infloran). Average nucleotide identity (ANI) analysis also supported these findings (100.00% nucleotide identity, Figure S5B). These data support the elevated *B. bifidum* relative abundances in our 16S rRNA gene profiling data (Figure 4A), including samples P8Z and P8ZA, which were collected at 41 and 50 days after supplementation had finished, indicating longer-term persistence of this strain (Figures 4F–4I).

*Bifidobacterium* represents a dominant genus in the full-term healthy breast-fed infant selectively fed by complex oligosaccharides (i.e., human milk oligosaccharides [HMOs]) within BM. However, the ability of *Bifidobacterium* to digest HMOs varies between species and strains of this genus.^31^^,^^32^ Thus, we analyzed *B. bifidum* genomes (our 5 isolates and Infloran strain) for the presence of genes involved in HMO utilization; all *B. bifidum* isolates contained specific genes involved in HMO utilization (Figure 4E), and mucin degradation genes that may aid gut persistence (Figure S5C). Notably, growth curves in whole BM (Figure 4C) confirmed that the *B. bifidum* Infloran strain utilized whole BM. Further phenotypic analysis indicated this strain was able to metabolize specific HMOs; 2-fucosyllactose (2′-FL) and Lacto-N-Neotetraose (LnNT), corresponding to genes *AfcA* and *BBgIII*, respectively, encoding for extracellular enzymes involved in their utilization (Figure 4D). Therefore, the ability to digest BM and HMOs in these predominantly BM-fed infants may correlate with higher rates of *Bifidobacterium* abundance.

Bacterial strains used as probiotics commonly lack antibiotic resistance genes. However, the high levels of antibiotic usage in the NICU may reduce abundance of supplemented strains in the preterm gut. Analysis of the *B. bifidum* Infloran strain genome indicated the presence of only the intrinsic *ileS* gene (associated with mupirocin resistance; Table S2). Minimum antibiotic concentration testing confirmed sensitivity to commonly prescribed antibiotics in NICUs (Table S2). These data are in agreement with the reduced relative abundance of *Bifidobacterium* in Bif/Lacto infants receiving antibiotics (Figure 3D) However, by giving the supplement twice daily (up to 34 weeks post-conceptual age), this may have aided rapid re-establishment after antibiotic treatment.

### Infants Receiving Oral Supplementation Show Differences in Metabolomic Profiles and Lower Fecal pH

**Figure 5:**
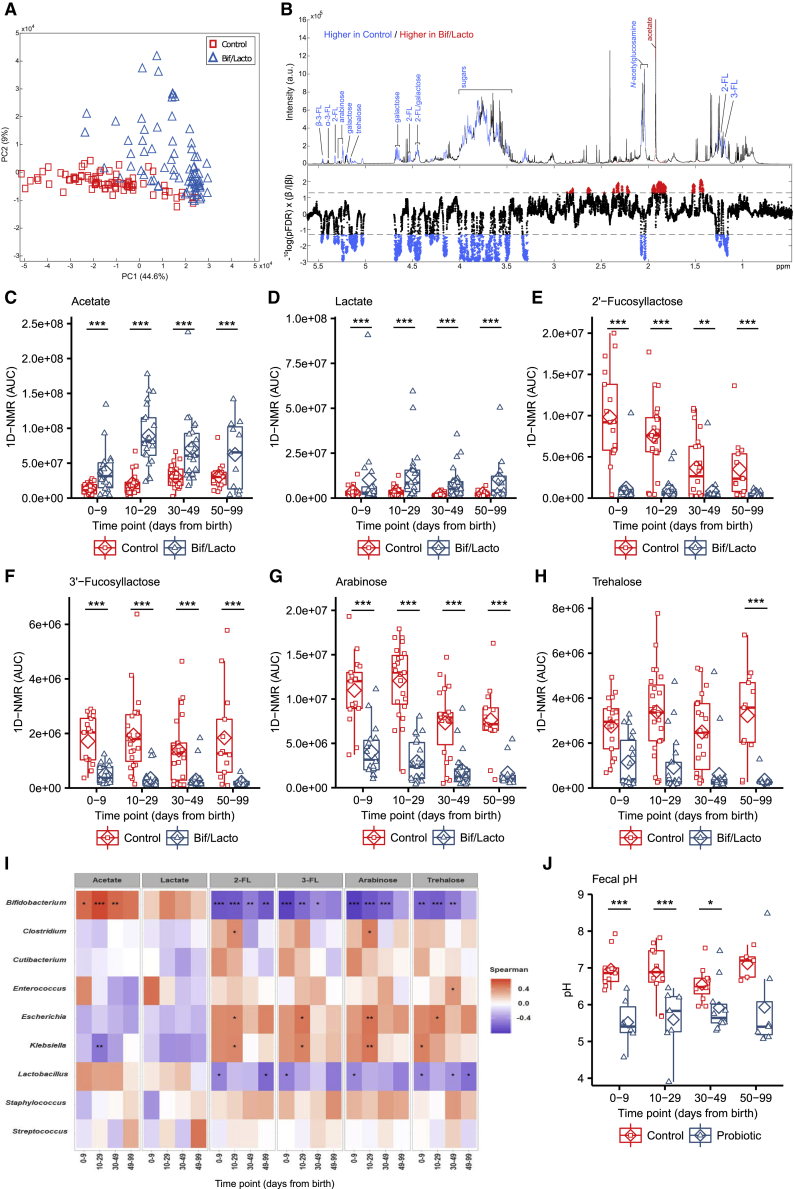
Metabolomic Profiling of Fecal Samples from the Bif/Lacto and Control Groups via ^1^H NMR Spectroscopy (A) Principal-component analysis (PCA) scores plot comparing the fecal metabolic profiles of the Bif/Lacto and control groups at all time points. (B) Discriminatory metabolites that contribute to the covariate-adjusted projection to latent structures-discriminant analysis (CA-PLS-DA) model comparing the fecal metabolic profiles of the Bif/Lacto and control infants adjusted for sampling age. Top panel: average ^1^H NMR spectrum from all samples indicating metabolites that are excreted in greater amounts by the Bif/Lacto infants (red) and those excreted in greater amounts by the control infants (blue). Bottom panel: Manhattan plot showing p values calculated for each variable in the multivariate model, corrected for multiple testing using the false discovery rate (allowing 5% false discoveries). Horizontal lines indicate cutoff values for the false discovery rate on the log_10_ scale. Blue points indicate metabolites significantly higher in the control feces and red points indicate those metabolites significantly higher in the Bif/Lacto feces. (C) Relative acetate concentration. (D) Relative lactate concentration. (E) Relative 2′-fucosyllactose (2-FL) concentration. (F) Relative 3′-fucosyllactose (3-FL) concentration. (G) Relative arabinose concentration. (H) Relative trehalose concentration. (I) Spearman correlation heatmap displaying main fecal metabolites (rows) versus the most abundant bacterial groups (columns). Red denotes positive correlation and blue denotes for negative correlation. For metabolite data (N = 0–9 days (control: n = 17, Bif/Lacto: n = 18); 10–29 days (control: n = 23, Bif/Lacto: n = 21); 30–49 days (control: n = 22, Bif/Lacto: n = 23); 50–99 days (control: n = 13, Bif/Lacto: n = 11)). (J) Group fecal sample pH (N = 0–9 days (control: n = 9, Bif/Lacto: n = 6); 10–29 days (control: n = 10, Bif/Lacto: n = 7); 30–49 days (control: n = 11, Bif/Lacto: n = 10); 50–99 days (control: n = 5, Bif/Lacto: n = 7)). Boxplots show group median and interquartile range, diamonds indicate the group mean, and individual points highlight individual infant samples. Asterisks represent p values: ∗p < 0.05, ∗∗p < 0.01, ∗∗∗p < 0.001. See also Figures S6 and S7 and Table S8.

Microbial metabolites are key molecules involved in microbe-microbe and microbe-host interactions.^33^ To define the “functional” impact of Bif/Lacto supplementation, ^1^H NMR spectroscopy was used to characterize the metabolomes of a subset of fecal samples (75 from Bif/Lacto group, and 81 from control group; all time points; n = 157), which were also profiled using 16S rRNA gene sequencing. A principal-component analysis (PCA) model (R^2^ = 53.6%) was built using these metabolic phenotypes, and clear biochemical variation was observed between the Bif/Lacto and control samples (Figure 5A). Pairwise orthogonal projection to latent structures-discriminant analysis (OPLS-DA) models constructed for each time point confirmed these metabolic differences throughout the study period (p < 0.01, Figure S6A). A covariate-adjusted PLS-DA (CA-PLS-DA) model comparing the fecal profiles at all sampling points and adjusted for sampling age showed that infants in the Bif/Lacto group excreted greater amounts of the short-chain fatty acid (SCFA) acetate (Figure 5C) and lower amounts of the sugars 2′-FL, 3-fucosyllactose (3′-FL), arabinose, and trehalose compared to those in the control group (Figures 5E–5H). Fecal lactate was also higher in Bif/Lacto infants compared to control infants (Figure 5D). Notably, the differences observed in fecal metabolites were maintained throughout the study period.

The relative abundance of *Bifidobacterium* was found to be significantly positively associated with fecal acetate and negatively associated with fecal 2′-FL, 3′-FL, arabinose, and trehalose (Figure 5I). Acetate and lactate are known metabolic by-products of *Bifidobacterium*, while 2′-FL and, 3′-FL are common components of HMOs, with certain *Bifidobacterium* strains (including Infloran Figure 4E) able to selectively metabolize these BM components.^32^ These results indicate that the higher relative abundance of *Bifidobacterium* in Bif/Lacto infants may correlate with the ability to metabolize HMOs, and with acetate and lactate generated as major end products.

To determine the impact of increased acetate and lactate on the infant gut environment, fecal pH was measured in a subset of infants (n = 74). At 0–9 days of age, fecal pH was 5.5 (SD = 0.7) in Bif/Lacto infants compared to pH 7.0 (SD = 0.5) in control infants (Table S9). These differences in fecal pH remained throughout the study (Figure 5J), and fecal pH was significantly negatively correlated with fecal acetate and lactate (Figures S6C and S6D) and the relative abundance of *Bifidobacterium* (Figure S6E). Comparing the relative bacterial abundance at species level, *B. bifidum* had a stronger negative correlation with fecal pH and positive correlation with fecal acetate and lactate (Figures S7A–S7C) compared to *B. breve*, the other main *Bifidobacterium* species present (Figures S7D–S7F). Metabolomic analysis on bacterial culture supernatant confirmed the strong acetate producing ability of the supplemented strain *B. bifidum* (Figure S7G).

## Discussion

Our results show that preterm infants supplemented with *B. bifidum* and *L. acidophilus* contain a fecal microbiota composition and environment more similar to a healthy full-term breast-fed infant.^10^^,^^11^ We determined that certain clinical practices and relevant external factors may positively or negatively influence the abundances of *Bifidobacterium* within the preterm infant gut microbiota.

Diet is a major driver of microbiota diversity, particularly the strong relationship between BM and *Bifidobacterium* abundance.^34^ Although both groups of preterm infants received high rates of BM, via maternal or donor milk, the low abundance of *Bifidobacterium* found in control infants indicates BM consumption itself (without supplementation) was not sufficient to encourage high levels of *Bifidobacterium*. We could not differentiate between (solely) donor versus maternal BM and impact on *Bifidobacterium* as within our study DBM was given to supplement the mother’s supply of BM. However, 16S rRNA gene profiles and sensitivity analyses indicate no consistent differences between microbiota composition (both for *Bifidobacterium* and pathobionts) in either Bif/Lacto infants or control infants between those fed mothers’ BM compared to those fed a combination of mothers’ BM and donor BM. Maternal to infant transmission of *Bifidobacterium* occurs in term infants^10^^,^^35^^,^^36^; however, the NICU environment and antibiotic treatment may limit establishment of parental *Bifidobacterium*, leaving infants susceptible to colonization by hospital-environmental bacteria.^2^^,^^37^ For the Bif/Lacto group, the combination of supplementation of early-life microbiota members and a known prebiotic food source, i.e., BM and HMOs, likely aided in enhanced persistence.^32^^,^^34^ Crucially, this synbiotic approach may allow the “right” bacterial strain matched to the appropriate nutritional environment. In this case, a *B. bifidum* strain with the genetic potential to metabolize HMOs and phenotypically shown to use these early-life dietary sources for growth. Interestingly, previous studies show *B. bifidum* secretes extracellular enzymes that facilitate cross-feeding of oligosaccharide degradation products among other *Bifidobacterium* species.^31^^,^^32^ Furthermore, *B. bifidum* strains are known to break down mucin, which may aid gut colonization.^38^ This microbiota supplementation strategy, including genomic and phenotypic analysis of the probiotic strain to confirm the ability to metabolize components of the early life diet, i.e., BM, is an important consideration for future studies.

Extremely low-birth-weight infants (<1,000 g) represented the most vulnerable cohort in this study and presented less abundance of genus *Bifidobacterium*, potentially due to several factors including, lengthened antibiotic courses,^39^ underdeveloped gut physiology (i.e., poorer gut motility, and thinner mucus layer^40^), and difficulties in establishing full enteral feeding.^41^ Indeed, previous clinical studies have had difficulties evaluating the beneficial effects of supplementation in this at-risk cohort of preterm infants,^42^ while others have seen a reduction in LOS but not in NEC.^42^^,^^43^ Notably, although extremely low-birth-weight Bif/Lacto infants had lower *Bifidobacterium* abundance than those infants weighing ≥1,000 g, supplementation in our study did enhance levels when compared to control infants. Thus, from an intervention strategy perspective, daily and prolonged supplementation may contribute to faster (re)establishment of *Bifidobacterium*, which may also promote colonization resistance against exogenous or resident pathogens in this particularly fragile preterm cohort.

*Bifidobacterium* abundance was not affected by delivery method with similarly high or low abundance of *Bifidobacterium*, respectively, within supplemented and control infants regardless of delivery method, either vaginal or caesarean. In contrast, vaginally delivered, full-term infants have been shown to have greater abundance of *Bifidobacterium* than infants born by caesarean delivery.^10^ Frequent antibiotic treatment in preterm infants may impact colonization by *Bifidobacterium* from the mother eliminating any early differences resulting from delivery method.

Rates of antibiotic prescription in preterm infants are remarkably high, ranging from 79% to 87% in extremely low-birth-weight (<1,000 g) preterm infants.^44^^,^^45^ Antibiotic treatment favors the establishment of antibiotic-resistant bacteria while indirectly eradicating highly susceptible microbiota members such as *Bifidobacterium.*^2^^,^^46^ Indeed, it appears that long- (but not short-) term antibiotic usage is correlated with reduced *Bifidobacterium* abundance (linking with low antimicrobial resistance genomic and phenotypic profiles in the supplemented *B. bifidum* strain), while potentially multidrug-resistant pathobionts such as *Klebsiella* and *Escherichia* abundance were unaffected by antibiotic duration. Recent research in infants correlated abundance of *Bifidobacterium* species (with/without supplementation) with a reduction in antimicrobial resistance genes and transferable elements.^17^^,^^47^ As Bif/Lacto infants had high relative abundance of *Bifidobacterium*, this may have contributed to reduce the reservoir of pathogens (i.e., *Staphylococcus*, *Escherichia*, and *Klebsiella*), which were prevalent in control infants, and which have previously been shown to harbor a large repertoire of AMR determinants (including in this cohort).^48^^,^^49^

Previous studies have indicated that, although preterm infants are particularly at risk of serious diseases with a bacterial cause (e.g., NEC and LOS), probiotic supplementation can reduce incidence.^13^^,^^14^ However, there has been variability in results, which may relate to the differences in strain(s) chosen, infant diet, or infant age. Notably, a recent clinical audit in the same NICU where the oral supplementation was given (i.e., Norfolk and Norwich University Hospital), indicated a >50% reduction in NEC rates and LOS when comparing 5-year epochs before and after introducing probiotic supplementation, with no episodes of probiotic “sepsis” indicated.^21^ While the processes leading to life-threatening conditions including NEC in preterm infants are complex, overgrowth of potentially pathogenic bacteria is thought to be a key factor.^48^^,^^50^^,^^51^ We show that supplemented preterm infants have lower relative abundance and overall prevalence of pathobionts including *Klebsiella* and *Escherichia*, which have previously been linked to NEC and LOS and links to a recent study that performed MinION shotgun metagenomics and AMR profiling on samples from this cohort.^48^ This may be due to direct inhibition through compounds secreted by *Bifidobacterium* (e.g., bacteriocins^52^), competition for space, and/or nutrient availability.

Low *Bifidobacterium* abundance has been consistently reported in preterm infants in NICUs without any supplementation use.^2^^,^^51^^,^^53^ This indicates that the primary finding of high proportions of bifidobacteria and associated changes in gut metabolites, in supplemented infants in this study is unlikely to be due to chance. Higher proportions of infants receiving probiotic supplements did receive mothers BM compared to a mix of BM and donor BM in controls. However, this did not result in any measurable difference (after multivariate and sensitivity analysis) in *Bifidobacterium* abundance or overall difference in microbiota composition. The HMOs in BM remain unaffected by the pasteurization and storage enabling donor milk to provide an equivalent substrate for the growth of bifidobacteria as BM.^54^ Concerns have been raised about the safety of using probiotic bacteria in vulnerable individuals.^55^ However, no adverse effects were observed to result from over 5 years of routine clinical use of probiotics used in this study.^21^

Differences in the gut environment were also indicated through our metabolomic analyses, highlighted by elevated abundance of acetate and lactate in feces from the Bif/Lacto group, which are known to be primary metabolic end products of HMO degradation by *Bifidobacterium*.^34^^,^^56^ Acetate and lactate have beneficial health effects enhancing defense functions in both host epithelial cells^57^ and mucosal dendritic cells.^58^ The lower fecal pH in Bif/Lacto group correlated with higher concentrations of these acids and higher abundance of *Bifidobacterium*, creating an acidic environment that may be less favorable for the growth of pathobionts.^57^^,^^59^^,^^60^

In summary, we have conducted a comprehensive observational study examining the beneficial impact of Bif/Lacto supplementation on the wider microbiota over time. Although previous studies have investigated aspects of this before,^17^, ^18^, ^19^, ^20^ this is the largest observational study to combine multiple factors: fecal microbiota composition analysis, metabolomics, fecal pH, whole-genome sequencing of supplemented probiotic strains, and fecal isolates to determine probiotic persistence, complemented by phenotypic testing. A key strength relates to the size and scope of the study, representing one of the largest reported longitudinal studies in preterm infants, where study cohorts were approximately matched by gestational age, sex, birth mode, and time points of sample collection, which are all factors that may significantly impact the microbiota, and thus conclusions obtained (see Limitations of Study below). Alongside the key microbiological findings of this study, we have also provided context for further trials focusing on clinical practice in NICU and suggestions for future intervention studies in this at-risk infant population. Providing maternal and donor BM may be required for successful persistence of *Bifidobacterium*, which may also contribute to the enhanced metabolic end-products such as acetate and lactate in the preterm gut. These products will play an important role in direct antagonism of potentially pathogenic microbes, and the maturation of immune cells in early life. This large-scale longitudinal observational multi-center-controlled study emphasizes the important role that targeted microbiota or probiotic supplementation plays in preterm infants, exerting beneficial modifications on preterm gut microbial communities, and metabolic end products.

### Limitations of Study

Our study has several limitations. First, this study was observational in nature and was not designed as a double-blinded, randomized controlled clinical study. One NICU recruited preterm infants receiving the Bif/Lacto oral supplementation, and three other NICUs recruited the control infants (not supplemented). Therefore, although the (UK) NICUs involved had comparable health care practices, there were some differences in feeding and antibiotics regimes between the two cohorts (and NICUs) that may impact microbiota profiles, with further alterations potentially due to the NICU environment (i.e., differences in nosocomial bacteria). This study recruited only seven infants who were exclusively formula fed (Table S1); therefore, it was not possible to assess the effect of routine supplementation in these infants. We could also not determine the impact of just DBS versus maternal BM due to the routine feeding practices in place (i.e., DBM only used to supplement, rather than replace). Future studies could carefully control for nutritional intake and perform analysis to understand how these diet differences may impact supplemented strain persistence and the wider preterm microbiota. In-depth analysis of the effects of different antibiotics regimes or antibiotic dosing on the premature infant gut microbiome was beyond of the scope of this work. This was due to the heterogeneity of timings and types of antibiotic used in routine clinical care, although controlling for antibiotic usage even in gold-standard clinical studies would also be problematic due to the at-risk nature of these preterm patients. As we used 16S rRNA profiling, this limits our analysis to relative abundance rather than absolute abundances of bacterial taxa; therefore, future studies could use a combination of qPCR (for supplemented strains, to determine colonization potential) in tandem with microbial load measurements. Another limitation is the fact that this was not a placebo-controlled trial and as such conclusions about (clinical) outcomes should be carefully interpreted.

## STAR★Methods

### Key Resources Table

**Table:** 

REAGENT or RESOURCE	SOURCE	IDENTIFIER
**Bacterial and Virus Strains**		

*Bifidobacterium breve* UCC2003 P16L	This paper	N/A
*Bifidobacterium breve* ACS-071-V-Sch8b P9P	This paper	N/A
*Bifidobacterium breve* 689b P74	This paper	N/A
*Bifidobacterium bifidum* S27 P74	This paper	N/A
*Bifidobacterium bifidum* BGN4 P15J	This paper	N/A
*Bifidobacterium bifidum* S17 P8Z	This paper	N/A
*Bifidobacterium bifidum* BGN4 P19K	This paper	N/A
*Bifidobacterium bifidum* S17 P36	This paper	N/A
*Bifidobacterium bifidum* BGN4 P8ZA	This paper	N/A
*Bifidobacterium bifidum* Infloran	This paper	N/A
*Lactobacillus acidophilus* Infloran	This paper	N/A

**Biological Samples**		

Preterm feces samples (Table S3)	This paper	N/A
Breast milk from voluntary donors	This paper	N/A

**Critical Commercial Assays**		

FastDNA Spin Kit for Soil	MP Biomedicals	Catalog number: 116560-200

**Deposited Data**		

Fastq. files from 16S rRNA gene sequencing	This paper	Accession number ENA: PRJEB31653
Fastq. files from whole genome sequencing	This paper	Accession number ENA: PRJEB31653

**Oligonucleotides**		

Primers used for 16 s RNA gene library (Table S4)	This paper	N/A

**Software and Algorithms**		

Trim galore version 0.4.3	Babraham Bioinformatics	http://www.bioinformatics.babraham.ac.uk/projects/trim_galore/
SILVA database version 123	German Network for Bioinformatics Infrastructure	https://www.arb-silva.de/documentation/release-123/
BLASTN version 2.2.25+	National Centre for Biotechnology Information	https://blast.ncbi.nlm.nih.gov/Blast.cgi?PAGE_TYPE=BlastDocs&DOC_TYPE=Download
R Studio version 1.1.463	R Studio	https://support.rstudio.com/hc/en-us/articles/206569407-Older-Versions-of-RStudio
ggplot2 R package version 3.1.0	R Documentation	https://www.rdocumentation.org/packages/ggplot2/versions/3.2.1
Prokka version 1.12	K Base Predictive Biology	https://kbase.us/applist/apps/ProkkaAnnotation/annotate_contigs/release?gclid=CjwKCAiAgqDxBRBTEiwA59eENxXKr8hh1HFvMYzy8BW-HKdD0r-ABTiXIOxSvV-5Ty2zyurO2VuewBoCv04QAvD_BwE
snp-dists version 0.2	Seemann, 2019^61^	https://github.com/tseemann/snp-dists
pyani version 0.2.7	Pritchard et al.^62^	https://github.com/widdowquinn/pyani
snp-sites version 2.3.3	Wellcome Sanger Institute	https://github.com/sanger-pathogens/snp-sites
Barrnap version 0.7	Institut Pasteur	http://bioweb.pasteur.fr/packages/pack@barrnap@0.7/
iTOL version 4.2	Letunic and Bork^63^	https://itol.embl.de/
RAxML version 8.2.10	Stamatakis^64^	https://cme.h-its.org/exelixis/web/software/raxml/
Snippy version 4.0	Biowulf	https://hpc.nih.gov/apps/snippy.html
Roary version 3.12.0	Wellcome Sanger Institute	https://sanger-pathogens.github.io/Roary/
MATLAB 9.4	MathWorks	https://uk.mathworks.com/campaigns/products/trials.html?gclid=CjwKCAiAgqDxBRBTEiwA59eEN25L0AauzlrTvocPJmLHSa2_FsOhEKfP_b8wDG5z5XQzVmG_uhjoGhoCMhsQAvD_BwE&ef_id=CjwKCAiAgqDxBRBTEiwA59eEN25L0AauzlrTvocPJmLHSa2_FsOhEKfP_b8wDG5z5XQzVmG_uhjoGhoCMhsQAvD_BwE:G:s&s_kwcid=AL!8664!3!252706741125!b!!g!!%2Bdownload%20%2Bmatlab&s_eid=ppc_6588248002&q=+download%20+matlab
Topspin 3.6	Bruker BioSpin	https://www.bruker.com/service/support-upgrades/software-downloads/nmr/free-topspin-processing/nmr-topspin-license-for-academia.html
ComplexHeatmap package version 1.18.1	Bioconductor	https://bioconductor.org/packages/release/bioc/html/ComplexHeatmap.html
Vegan package version 2.5-4	Torondel et al.^65^	https://cran.r-project.org/web/packages/vegan/index.html
Megan6	Bağcı et al.^66^	https://software-ab.informatik.uni-tuebingen.de/download/megan6/welcome.html
Phyloseq package version 1.24.2	McMurdie and Holmes^67^	https://bioconductor.org/packages/release/bioc/html/phyloseq.html

**Other**		

LNnT (donated from Glycom)	Glycom	https://www.glycom.com/
2′FL (donated from Glycom)	Glycom	https://www.glycom.com/
Zirconium beads 1 mm diameter	BioSpec Products	Cat. No. 11079110z
Infloran	Desma Healthcare	https://www.desmahealthcare.com/products

### Resource Availability

#### Lead Contact

Further information and requests for resources and reagents should be directed to and will be fulfilled by the Lead Contact, Lindsay J. Hall (Lindsay.Hall@quadram.ac.uk).

#### Materials Availability

This study did not generate new unique reagents.

#### Data and Code Availability

The 16S rRNA and WGS datasets generated during this study are available at the European Nucleotide Archive: PRJEB31653. The accession numbers for the European Nucleotide Archive sequence data reported in this paper are included in Data S1 and Table S3. The code (R scripts) are available at: https://github.com/dalbymj/BAMBI-Paper-Files.

### Experimental Model and Subject Details

#### Exclusion and inclusion criteria (human cohorts)

All subjects recruited in this study were premature infants born at gestational age ≤ 34 weeks, and resident in the same NICU for study duration. Infants diagnosed with advanced stages of necrotizing enterocolitis or severe congenital abnormalities, were excluded from the study.

Preterm infants were recruited from four different NICUs across England, UK (between 2013-2017); Norfolk and Norwich University Hospital (NNUH) enrolled the Bif/Lacto group, and Rosie Hospital, Queen Charlotte’s and Chelsea Hospital, and St Mary’s Hospital recruited Control group infants. All NICUs had comparable health care practices including antibiotic (short treatment > 3 days; long treatment > 3 days) and antifungal policies. To minimize the influence of confounding factors Bif/Lacto versus Control groups included similar sex ratios and delivery mode (i.e., Caesarean-section or vaginal delivery) (Table S1). We preferably selected preterm infants from both study groups which had received their mother’s own breast milk or donor breast milk; the majority were exclusively breastfed or received donor breast milk (78% in Bif/Lacto Group and 76% Control Group), mixed fed with a combination of breastmilk, formula or donor breast milk (20% Bif/Lacto Group and 20% Control Group), and exclusively formula fed (2% Bif/Lacto Group, and 4% Control Group).

#### Ethical approval

Fecal collection from NNUH and Rosie Hospital was approved by the Faculty of Medical and Health Sciences Ethics Committee at the University of East Anglia (UEA), and followed protocols laid out by the UEA Biorepository (License no: 11208). Fecal collection for Queen Charlotte’s and Chelsea Hospital and St Mary’s Hospital was approved by West London Research Ethics Committee (REC) under the REC approval reference number 10/H0711/39. In all cases, doctors and nurses recruited infants after parents gave written consent.

#### Human study design

Two distinct preterm groups were recruited: 1) Bif/Lacto Group who routinely received oral *Bifidobacterium* and *Lactobacillus* supplementation (n = 101 infants), and 2) Control Group infants who did not receive supplementation (n = 133 infants). Infants in the Bif/Lacto group were prescribed daily oral supplementation of 10^9^ colony forming units (CFU) of *Bifidobacterium bifidum* and 10^9^ CFU of *Lactobacillus acidophilus* (Infloran®, Desma Healthcare, Chiasso, Switzerland). This supplementation was given twice daily in a divided dose and commenced with the first enteral colostrum/milk feed (usually day 1 postnatal). Oral supplementation was normally administered until 34 weeks post-conceptual age, with the exception of very low birth weight infants (< 1500 g) who received it until discharge. Half a capsule of Infloran (125 mg) was dissolved in 1 mL of expressed breastmilk and/or sterile water, and this dose was given twice daily (250mg/total/day) to the infant via nasogastric tube.

Time points of sample collection for this study included 0-9 days, 10-29 days, 30-49 days, 50-99 days. Research nurses collected clinical data from hospital databases and clinical notes including; gestational age, delivery mode, antibiotic courses received, and dietary information (Table S3).

### Methods Details

#### DNA extraction of preterm stool samples

FastDNA Spin Kit for Soil (MP) was used to extract DNA from preterm feces following manufacturer instructions, with extended 3 min bead-beating. DNA concentration and quality were quantified using a Qubit® 2.0 fluorometer (Invitrogen).

#### 16S rRNA gene sequencing of fecal samples

16S rRNA region (V1-V2) primers were used for library construction. Table S4 details primers sequences used. This set of primers allowed the amplification of one 16S rRNA gene sequencing library containing 96 different samples. PCR conditions used were; cycle of 94°C 3 min and 25 cycles of 94°C for 45 s, 55°C for 15 s and 72°C for 30 s. Sequencing of the 16S rRNA gene libraries was performed using Illumina MiSeq platform with 300 bp paired end reads.

Raw reads were filtered through quality control using trim galore (version 0.4.3), minimum quality threshold of phred 33, and minimum read length of 60 bp. Reads that passed threshold were aligned against SILVA database (version: SILVA_132_SSURef_tax_silva) using BLASTN (ncbi-blast-2.2.25+; Max e-value 10e-3) separately for both pairs. After performing BLASTN alignment, all output files were imported and annotated using the paired-end protocol of MEGAN6 on default Lowest Common Ancestor (LCA) parameters^66^.

The number of reads required to obtain a reliable representation of the microbiota in each sample was assessed by generating rarefaction curves using the vegan package in R. Rarefaction curves were used to identify 20,000 as the minimum number of reads in a sample at which the number of new genus appearing plateaued. Samples contained an average of less than ten genera and so failing to detect even one or two genera from a sample would significantly alter the composition of the sample microbiota. The 16S rRNA gene sequence data was subsampled to an even depth of 20,000 read using the phyloseq package (version 1.24.2), which removed 63 samples with fewer than 20,000 reads. Additionally, the 16S rRNA gene sequence data with samples with less than 20,000 reads removed was normalized using two alternative methods, mean log-transformation or variance stabilization, using the Deseq2 package in R. The number of infants with samples in the two groups at each time point after normalization were Bif/Lacto: 0-9 days = 55; 10-29 days = 83; 30-49 days = 48; 50-99 days = 39 and Control: 0-9 days = 93; 10-29 days = 99; 30-49 days = 53; 50-99 days = 32. Sample details with proportion of reads assigned to each bacterial genus can be found in Table S6 and species in Table S7. R Studio version 1.1.463 including the ggplot2 R package version 3.1.0 was used for the analysis of microbiota sequence data and generation of figures.

#### Genomic DNA extraction from bacterial isolates

We isolated the strains present in the oral supplementation (i.e., *Bifidobacterium bifidum* and *Lactobacillus acidophilus)* as well as additional *Bifidobacterium* isolates from infant samples. Overnight pure cultures in Brain Heart Infusion Broth (BHI) were harvested for phenol-chloroform DNA extraction. Bacterial pellets were resuspended in 2 ml 25% sucrose in 10 mM Tris and 1 mM EDTA at pH 8. Cells were subsequently lysed adding 50 μl 100 mg/ml lysozyme (Roche) and incubating at 37°C for 1 h. 100 μl 20 mg/ml Proteinase K (Roche), 30 μl 10 mg/ml RNase A (Roche), 400 μl 0.5 M EDTA (pH 8.0) and 250 μl 10% Sarkosyl NL30 (Fisher) was added into the lysed bacterial suspension, incubated 1 h on ice and left overnight at 50°C. Next, washes of phenol-chloroform-isoamyl alcohol (PCIA, Sigma) using 15 ml gel-lock tubes (QIAGEN), with E Buffer (10mM Tris pH 8 (Fisher Scientific, UK)) added to sample to a final volume of 5 ml, mixed with 5 mL of PCIA (Sigma) and centrifuged for 15 min at 4000 rpm. The CIA step was repeated three times, after which the final aqueous phase was transferred into sterile Corning ™ 50 mL centrifuge tubes, and 2.5 volumes of ethanol (VWR Chemicals, USA) added, incubated for 15 min at −20°C, and centrifuged 10 min at 4000 rpm and 4°C. Finally, the pellet was washed twice with 10 mL of 70% ethanol and centrifuged at 4000 rpm for 10 min, dried overnight, and re-suspended in 300 μl of E Buffer.

#### Whole genome sequencing of bacterial isolates

DNA from pure bacterial cultures was sequenced at Wellcome Trust Sanger Institute using 96-plex Illumina HiSeq 2500 platform to generate 125 bp paired end reads as described previously^68^. Genome assembly was performed by the sequencing provider using the assembly pipeline described by Page et al., 2016^69^. Next, genome assemblies were annotated using Prokka (version 1.12). We predicted the 16S rRNA gene from the whole genome data using barrnap (version 0.7) and compare it to with existing 16S rRNA gene sequences. Single Nucleotide Polymorphisms (SNPs) were identified using Snippy (version 4.0) by mapping assembled contigs to annotated reference Infloran *B. bifidum* strain to reconstruct SNP phylogeny of six *B. bifidum* strains^70^.

To construct a phylogeny of 10 *Bifidobacterium* strains, we used pangenome pipeline Roary (version 3.12.0) to build a core gene alignment (87 core genes, with options -e -n otherwise default), followed by snp-sites (version 2.3.3) to call SNPs (6,202 SNPs in total)^69^^,^^71^. We used the SNP site-alignments obtained from both reference-based and core-gene alignment approaches to infer Maximum Likelihood (ML) phylogenies using RAxML (version 8.2.10) with GTR+ nucleotide substitution model at 100 permutations conducted for bootstrap convergence test^63^. The ML tree reconstructed was with the highest likelihood out of 5 runs (option -N 5). Pairwise SNP distances were calculated and compared using snp-dists (version 0.2)^61^. Pairwise Average Nucleotide Identity (ANI) was computed and graphed using module pyani (version 0.2.7)^62^. Web tool iTOL version 4.2 was used to visualize and annotate ML trees^63^.

#### Minimal Inhibitory Concentration analysis

The microdilution method was used to test Minimal Inhibitory Concentration (MIC) of the probiotic strains (*B. bifidum*) against routinely prescribed antibiotics; benzylpenicillin, gentamicin, and meropenem. Serial twofold dilutions of antibiotics in MRS medium (Difco) and 10 μL from fresh overnight culture were incubated for 24 h at 37°C under anaerobic conditions. Cell density was monitored using a plate reader (BMG Labtech, UK) at 595 nm. MICs were determined as the lowest concentration of antibiotic inhibiting any bacterial growth, with tests performed in triplicate.

#### Gene search using BLAST

Genomes from *B. bifidum* Infloran strain and five other *B. bifidum* isolates were searched for genes involved in utilization of human milk oligosaccharides, and mucin degradation. Nucleotide sequences of genes of interest were extracted from National Centre of Biotechnology Information (NCBI). Table S5 summarizes genes analyzed and publication source. BLAST alignment (ncbi-blast-2.2.25) was performed using a filtering criteria of 80% coverage and 80% identity.

#### Breast milk and HMOs utilization study

Growth kinetics of the Infloran isolate *B. bifidum* and control type strains *B. longum* subsp. *infantis* DSM 20088 and *B. breve* DSM 20213 in breast milk and individual HMOs (LNnT or 2′FL) were performed. Isolates were grown overnight in RCM (Oxoid) then subcultured into modified MRS (Difco) with breast milk (pooled from four different mothers, collected at eight different time-points, 1% w/v), or individual HMOs (2% w/v)^32^. Growth kinetics were measured every 15 minutes for 48 hours using a microplate spectrophotometer (Tecan Infinite F50).

#### Metabolomic analysis 1D-NMR and 2D-NMR

A subset of 157 paired fecal samples (75 from Bif/Lacto group, and 81 from Control group) were analyzed by standard one-dimensional (1D) ^1^H NMR spectroscopy using a Bruker 600 MHz spectrometer operating at 300 K. Fecal samples were chosen for metabolic profiling pragmatically based on remaining sample quantity after previous analyses. Feces (50 mg) were combined with 700 μL of phosphate buffer (pH 7.4; 100% D_2_O) containing 1 mmol/L of 3-trimethylsilyl-1-[2,2,3,3-^2^H_4_] propionate (TSP), and 10 zirconium beads (1 mm diameter) (BioSpec Products). Samples were homogenized using a Precellys bead beater (Bertin) with 2 cycles of 40 s at 6,500 Hz speed, centrifuged at 14,000 *g* for 10 min and the supernatant was transferred to NMR tubes. 1D NMR spectra were acquired for each sample using a nuclear overhauser effect pulse sequence for water suppression as described by Beckonert and colleagues^72^). Spectra were automatically phased and calibrated to the TSP reference using Topspin 3.6 (Bruker BioSpin). Spectra were imported into MATLAB 9.4 (R2018a), redundant spectral regions (those arising from TSP and imperfect water suppression) were removed, and the spectral profiles were normalized using a probabilistic quotient method.

#### pH measurement of the fecal samples

The pH of a randomly selected subset of fecal samples used in the metabolomics analysis (39 samples from the Bif/Lacto Group, and 39 samples from the Control Group) was assessed. Fifty mg of fecal sample was added 1 mL of sterile water, vortexed and measured using a glass electrode pH meter (Martini Mi151).

### Quantification and statistical analysis

16S rRNA gene sequencing data was analyzed using NMDS (Non-metric multidimensional scaling) plots generated with a Bray-Curtis dissimilarity calculation in R Studio using with the vegan package version 2.5-4 using code adapted from^65^. Permutational MANOVA in the Adonis function of the vegan R package version 2.5-4 was used to determine significant differences between NMDS community structure. Heatmaps were generated using the ComplexHeatmap package version 1.18.1 and clustered using a Bray-Curtis dissimilarity calculation. Genus number, Shannon diversity, and Inverse Simpson diversity were calculated using the vegan package version 2.5-4. Statistically significant differences in genus and species abundance were determined using a Kruskal-Wallis test corrected for false discovery rate (FDR < 0.05). To account for differences in the proportion of infants receiving only mother’s breast milk and long-duration antibiotics PERMANOVA was carried out to analyze the effects on the relative abundance of the individual genera of *Bifidobacterium*, *Klebsiella*, and *Escherichia*. Sensitivity analysis was carried out by selecting and either comparing only those samples collected when the infant was receiving only mother’s breast milk or only samples from infants that had received short duration antibiotics. Within these differences in overall microbiota composition were compared between Bif/Lacto and Control groups using NMDS and PERMANOVA as detailed previously while individual differences in genus relative abundance were determined using a Kruskal-Wallis test corrected for false discovery rate (FDR < 0.05). The distribution of continuous variables was tested using the Shapiro–Wilk test with a significance threshold of < 0.05 and the appearance of boxplots, Quantile–Quantile plots, and histograms also considered. For continuous variables, according to the distribution, either t test or Wilcoxon rank-sum test was used to test the significance of difference between groups. Differences in percentage prevalence of individual bacterial genera were tested using tested using Fisher’s exact test. A p value of less that 0.05 was considered statistically significant for all tests.

1D-NMR data analysis was performed using principal components analysis (PCA), orthogonal projection to latent structures discriminant analysis (OPLS-DA) and covariate-adjusted-projection to latent structures-discriminant analysis (CA-PLSDA) using in-house scripts. Pairwise OPLS-DA models (Bif/Lacto versus Control) were constructed for each sampling point and for all sampling points combined. Here, the complete spectral data points (metabolic profile) served as the predictors (X variables) and class membership (Bif/Lacto versus Control) served as the response (Y) variable. The predictive ability (Q^2^Y) of the models were calculated using a 7-fold cross-validation approach and the validity of the Q^2^Y values were assessed through permutation testing (100 permutations). A CA-PLS model was also built using the fecal profiles from all sampling points and the model was adjusted for sampling age.

Additional two-dimensional (2D) ^1^H-^1^H NMR spectroscopy was performed on two selected fecal samples to assist with metabolite identification. Conventional 2D NMR spectra were acquired using homonuclear correlation spectroscopy (COSY) and heteronuclear single quantum coherence spectroscopy (HSQC) experiments with water suppression to assist with structural elucidation.

## Supplemental Information

Document S1. Figures S1–S7, Table S1, and Data S1–S7

Table S2. AMR Gene Analysis and Phenotypic Characterization of *Bifidobacterium bifidum* (Infloran)

Table S3. Metadata for All Infant Samples

Table S4. Primer Sequences for Amplifying V1-V2 Region of the 16S rRNA Gene Using MiSeq Illumina

Table S5. *Bifidobacterium bifidum* Human Milk Oligosaccharides and Mucin Degradation Genes

Table S6. Genus Relative Abundance as Percentages in Each Preterm Infant Sample Analyzed

Table S7. Species Relative Abundance and Percentages in Each Infant Sample.

Table S8. Metabolites Detected with 1D-NMR

Table S9. pH Measurements Bif/Lacto and Control Group

Table S10. Multivariate Analysis Using PERMANOVA to Test the Variation in Microbiota Composition Explained by Each Factor

Table S11. Multivariate Analysis Using PERMANOVA to Test the Variation in Microbiota Composition Explained by Neonatal Intensive Care Unit

Table S12. Multivariable Analysis Using PERMANOVA with a Euclidian Distance Matrix and Multilevel Pairwise Comparison Bonferroni Correction for Influence of Group, Diet, and Antibiotic Duration on the Relative Abundance of *Bifidobacterium*, *Klebsiella*, and *Escherichia*

Document S2. Article plus Supplemental Information
