# Supplementary material for: Microbiota Supplementation with Bifidobacterium and Lactobacillus Modifies the Preterm Infant Gut Microbiota and Metabolome: An Observational Study
Source: Cell Rep Med. 2020 Aug 25;1(5):100077. doi: 10.1016/j.xcrm.2020.100077 (PMC7453906; doi:10.1016/j.xcrm.2020.100077)
Supplement: Document S1. Figures S1–S7, Table S1, and Data S1–S7 [file mmc1.pdf]

Cell Reports Medicine, Volume *1*

Supplemental Information

Microbiota Supplementation with *Bifidobacterium* and *Lactobacillus* Modifies the Preterm Infant Gut Microbiota and Metabolome: An Observational Study

Cristina Alcon-Giner, Matthew J. Dalby, Shabhonam Caim, Jennifer Ketskemety, Alex Shaw, Kathleen Sim, Melissa A.E. Lawson, Raymond Kiu, Charlotte Leclaire, Lisa Chalklen, Magdalena Kujawska, Suparna Mitra, Fahmina Fardus-Reid, Gustav Belteki, Katherine McColl, Jonathan R. Swann, J. Simon Kroll, Paul Clarke, and Lindsay J. Hall

Table S1: Summary statistics for preterms recruited in the study. Related to Figure 1.

	Group		
	**Control**	**Bif/Lacto**	**p-value**
**n**	**133**	**101**	
**Sex (n (%))**			
Female	75 (56.4)	45 (44.6)	0.096
Male	58 (43.6)	56 (55.4)	
**Delivery (n (%))**			
Vaginal	61 (45.9)	46 (45.5)	1
Cesarean	72 (54.1)	55 (54.5)	
**Birthweight in grams (mean (SD))**	1146.02 (337.19)	1127.17 (311.97)	0.662
**Gestational age in weeks (mean (SD))**	28.35 (2.29)	28.55 (2.77)	0.545
**Length of NICU stay (days) (mean (SD))**	45.86 (22.79)	53.06 (32.71)	0.055
**Length of antibiotics (n (%))**			
Long	25 (18.8)	42 (41.6)	0.001
Short	94 (70.7)	54 (53.5)	
None	13 (9.8)	5 (5.0)	
NA	1 (0.8)	0 (0.0)	
**Hospital (n (%))**			
Norfolk and Norwich	-	101 (100.0)	
St Mary's	65 (48.9)	-	
Queen Charlotte's	54 (40.6)	-	
Addenbrookes	14 (10.5)	-	
**Total diet composition (n (%))**			
Breast milk	36 (27.1)	70 (69.3)	<0.001
Breast milk + Donor breast milk	68 (51.1)	7 (6.9)	
Breast milk + Formula	13 (9.8)	14 (13.9)	
Breast milk + Donor breast milk + Formula	13 (9.8)	5 (5.0)	
Donor milk	0 (0.0)	1 (1.0)	
Formula	3 (2.3)	4 (4.0)	

n = number of infants.

SD = standard deviation.

Length of antibiotics: Short (< 3days); Long (> 3 days).

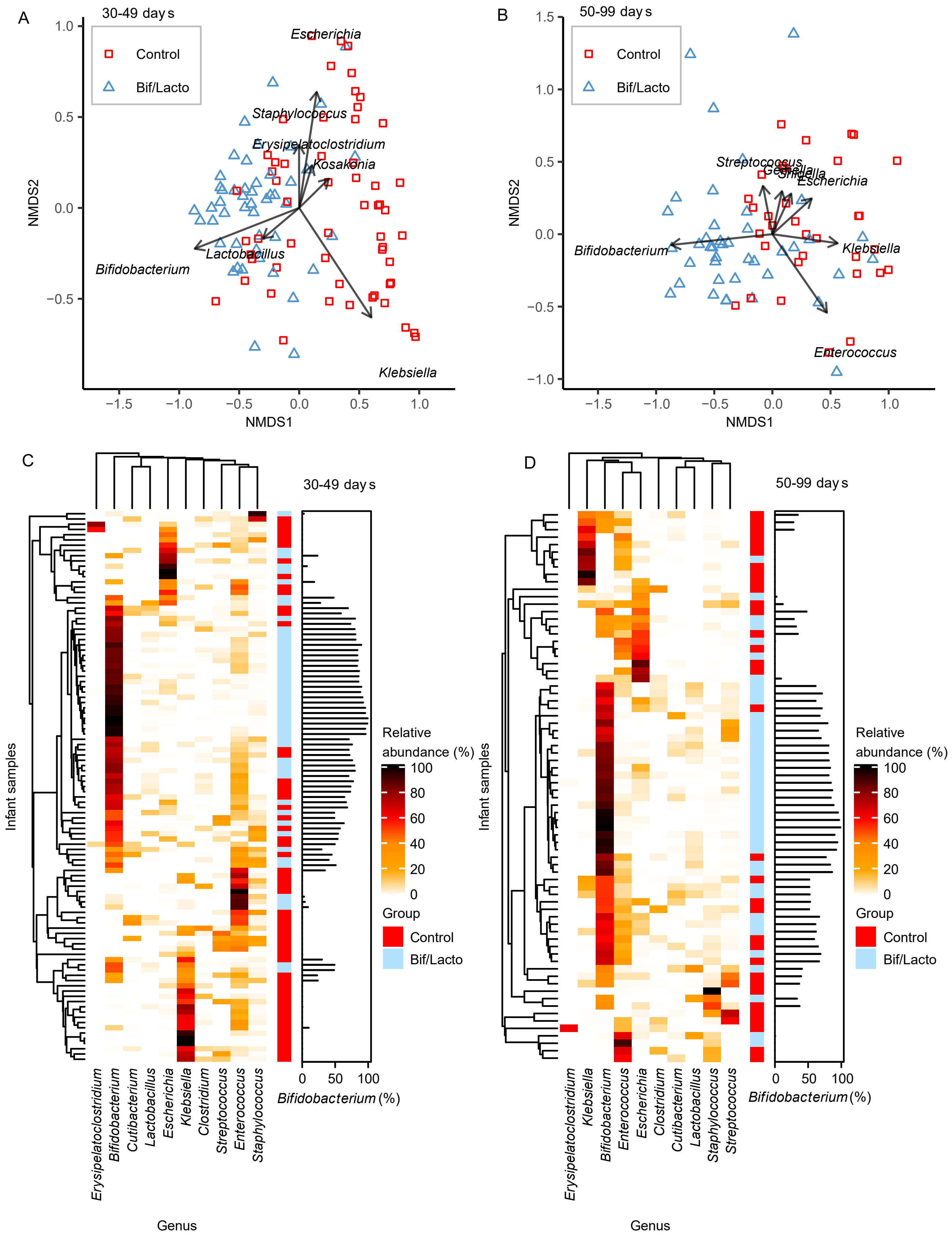
A
30-49 days
Control
Bif/Lacto
Escherichia
Staphylococcus
Erysipelatoclostridium
Kosakonia
Lactobacillus
Bifidobacterium
Klebsiella
NMDS1
NMDS2
1.0
0.5
0.0
−0.5
−1.5
−1.0
−0.5
0.0
0.5
1.0
B
50-99 days
Control
Bif/Lacto
Streptococcus
Gemella
Shigella
Escherichia
Bifidobacterium
Klebsiella
Enterococcus
NMDS1
NMDS2
1.5
1.0
0.5
0.0
−0.5
−1.0
C
30-49 days
Infant samples
Relative abundance (%)
100
80
60
40
20
0
Group
Control
Bif/Lacto
Erysipelatoclostridium
Bifidobacterium
Cutibacterium
Lactobacillus
Escherichia
Klebsiella
Clostridium
Streptococcus
Enterococcus
Staphylococcus
0
50
100
Bifidobacterium (%)
Genus
D
50-99 days
Infant samples
Relative abundance (%)
100
80
60
40
20
0
Group
Control
Bif/Lacto
Erysipelatoclostridium
Klebsiella
Bifidobacterium
Enterococcus
Escherichia
Clostridium
Cutibacterium
Lactobacillus
Staphylococcus
Streptococcus
0
50
100
Bifidobacterium (%)
Genus

Figure S1 related to Figure 1. Clustering of samples and genus composition.

(A) NMDS plot clustered with a Bray-Curtis dissimilarity of infant fecal microbiota similarity at 30-49 days (Bif/Lacto: n = 50, Control: n = 63).

(B) NMDS plot clustered with a Bray-Curtis dissimilarity of infant fecal microbiota similarity at 50-99 days (Bif/Lacto: n = 43, Control: n = 35).

Arrows indicate bacterial genera driving the separation of points on the NMDS plots.

(C) Heatmap with the ten genera with highest relative abundance at 30-49 days of age.

(D) Heatmap with the ten genera with highest relative abundance at 50-99 days of age.

Heatmap rows were clustered using Bray-Curtis dissimilarity. Side bar plots show the proportional abundance of *Bifidobacterium* in each sample.

Related to Figure 1.

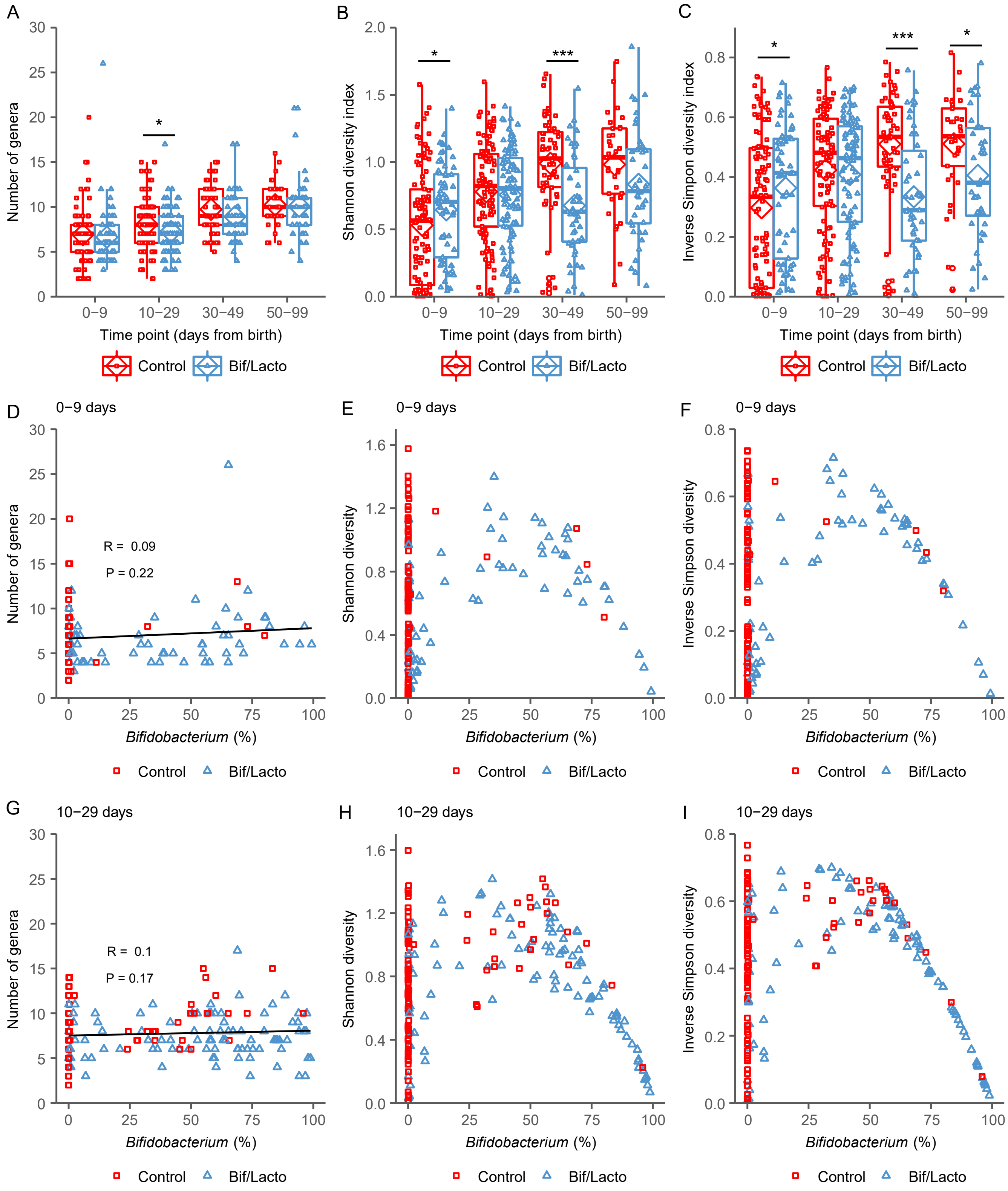

A
B
C
Number of genera
Shannon diversity index
Inverse Simpson diversity index
Time point (days from birth)
0–9
10–29
30–49
50–99
*
***
Control
Bif/Lacto
D
E
F
0–9 days
R = 0.09
P = 0.22
Number of genera
Shannon diversity
Inverse Simpson diversity
Bifidobacterium (%)
G
H
I
10–29 days
R = 0.1
P = 0.17

Figure S2 related to Figure 2. Microbiota diversity.

(A) Number of genera detected in each infant sample.

(B) Shannon diversity index of each infant sample.

(C) Inverse Simpson diversity index of each infant sample.

(D) Number of genera against *Bifidobacterium* abundance at 0-9 days.

(E) Shannon diversity against *Bifidobacterium* abundance at 0-9 days.

(F) Inverse Simpson diversity against *Bifidobacterium* abundance at 0-9 days.

(G) Number of genera against *Bifidobacterium* abundance at 10-29 days.

(H) Shannon diversity against *Bifidobacterium* abundance at 10-29 days.

(I) Inverse Simpson diversity *Bifidobacterium* abundance at 10-29 days.

Individual points show each infant sample, the diamond indicates the group mean, box plots show median and interquartile ranges.

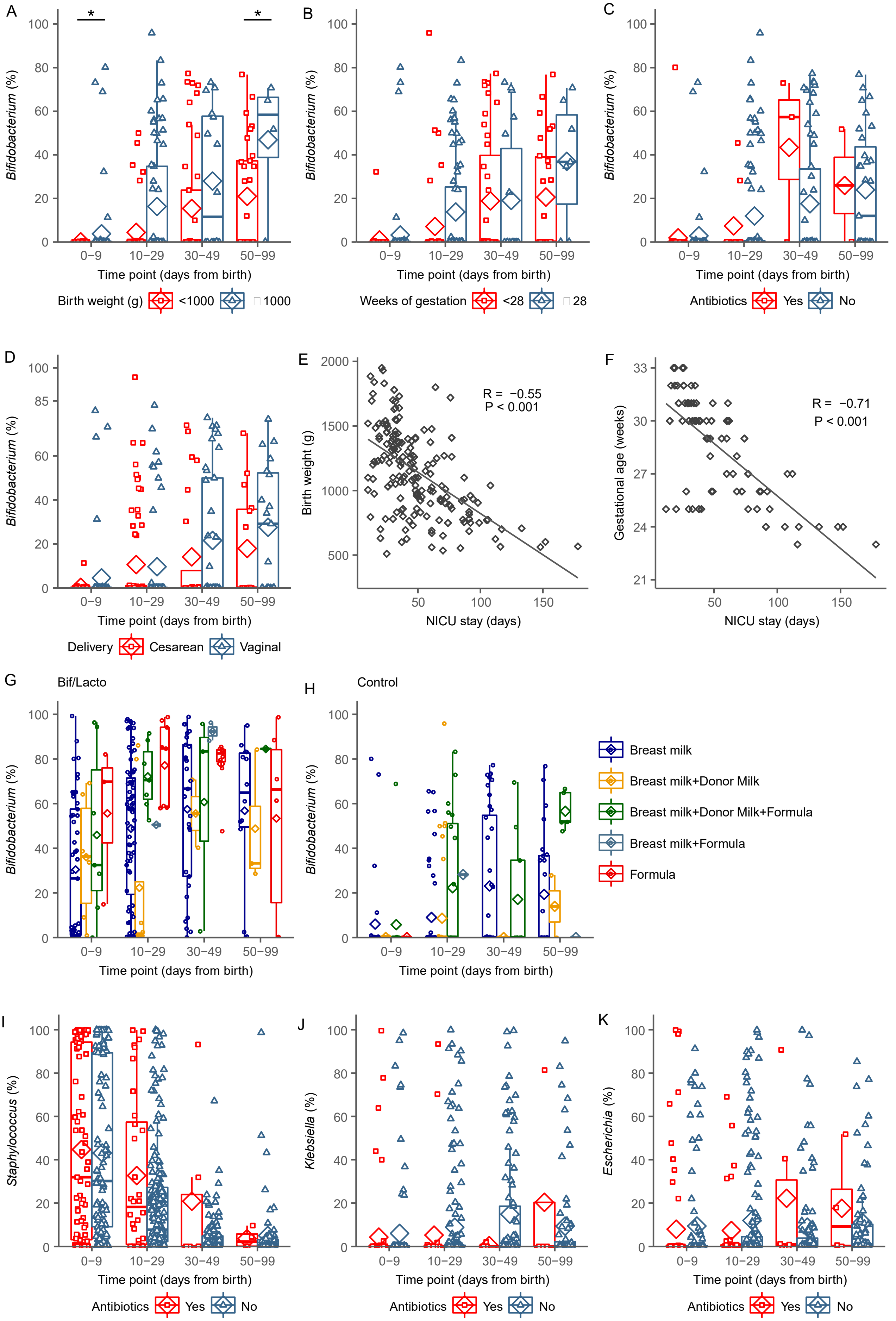
A
B
C
*
*
Bifidobacterium (%)
0–9
10–29
30–49
50–99
Time point (days from birth)
Birth weight (g)
<1000
□1000
Weeks of gestation
<28
□28
Antibiotics
Yes
No
D
Delivery
Cesarean
Vaginal
E
R = −0.55
P < 0.001
Birth weight (g)
NICU stay (days)
F
R = −0.71
P < 0.001
Gestational age (weeks)
NICU stay (days)
G
Bif/Lacto
H
Control
Breast milk
Breast milk+Donor Milk
Breast milk+Donor Milk+Formula
Breast milk+Formula
Formula
I
J
K
Staphylococcus (%)
Klebsiella (%)
Escherichia (%)

Figure S3 related to Figure 2: Effects of birthweight, antibiotics, delivery mode and diet on *Bifidobacterium*.

(A) *Bifidobacterium* abundance in Control infants by birth weight.

(B) *Bifidobacterium* abundance in Control infants by gestational age.

(C) *Bifidobacterium* abundance in Control infants receiving antibiotics at the time of sample collection.

(D) *Bifidobacterium* abundance in Control infants delivered by caesarean or vaginal birth.

(E) Birth weight correlated with length of stay in NICU in all infants.

(F) Gestational age correlated with length of stay in NICU in all infants.

(G) Bif/Lacto group *Bifidobacterium* abundance by diet group.

(H) Control group *Bifidobacterium* abundance by diet group.

(I) *Staphylococcus* abundance in all infants receiving antibiotics at the time of sample collection.

(J) *Klebsiella* abundance in in all infants receiving antibiotics at the time of sample collection.

(K) *Escherichia* abundance in in all infants receiving antibiotics at the time of sample collection.

Individual points show each infant sample, the diamond indicates the group mean, box plots show median and interquartile ranges. Asterisks represent *p* values: $*P < 0.05$.

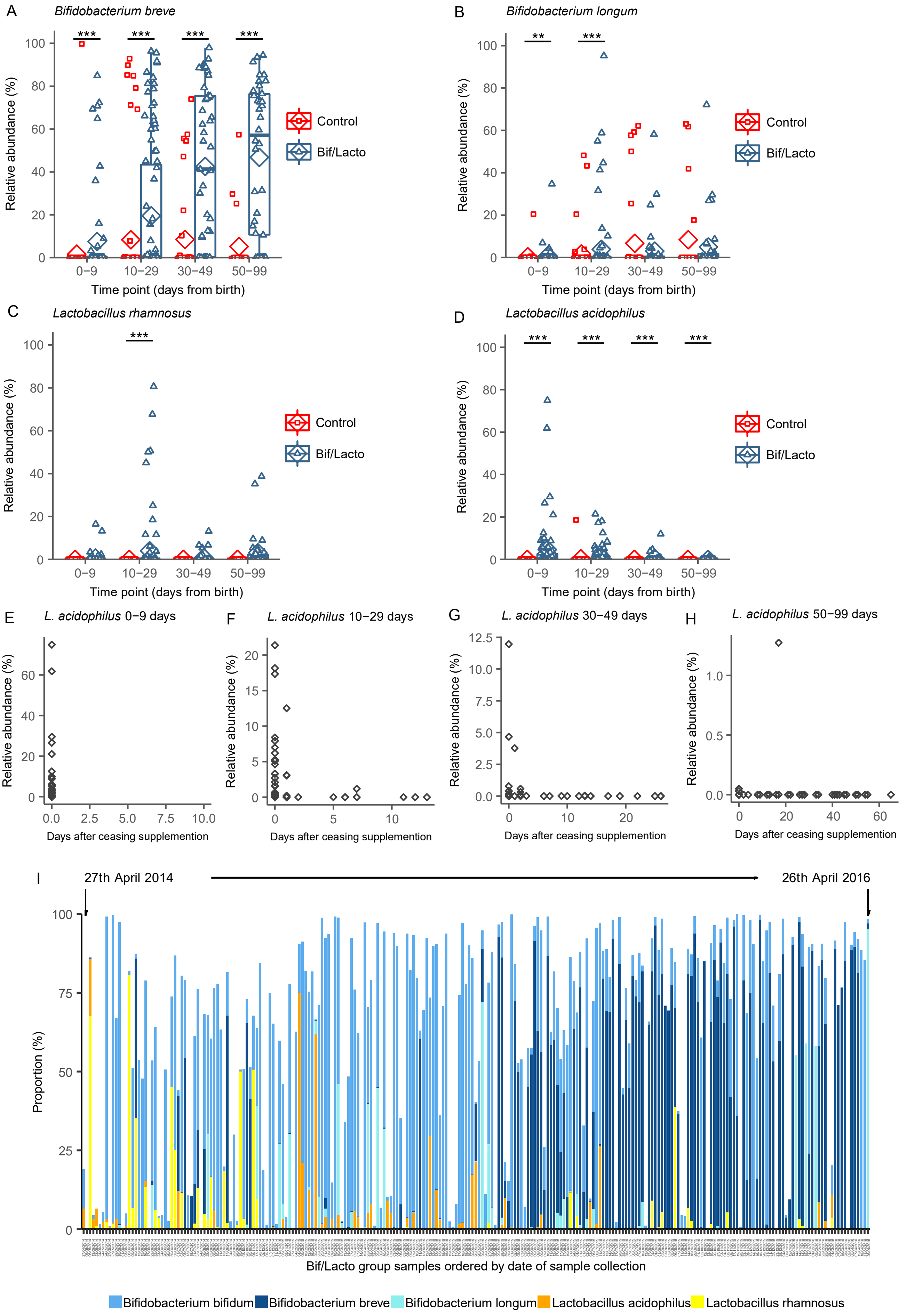
A
Bifidobacterium breve
B
Bifidobacterium longum
C
Lactobacillus rhamnosus
D
Lactobacillus acidophilus
***
**
Relative abundance (%)
Time point (days from birth)
0−9
10−29
30−49
50−99
Control
Bif/Lacto
E
L. acidophilus 0−9 days
F
L. acidophilus 10−29 days
G
L. acidophilus 30−49 days
H
L. acidophilus 50−99 days
Days after ceasing supplementation
I
27th April 2014
26th April 2016
Proportion (%)
Bif/Lacto group samples ordered by date of sample collection
Bifidobacterium bifidum
Bifidobacterium breve
Bifidobacterium longum
Lactobacillus acidophilus
Lactobacillus rhamnosus

Figure S4 related to Figure 4. *Bifidobacterium* species level abundance and over time.

(A) *Bifidobacterium breve* proportional abundance.

(B) *Bifidobacterium longum* proportional abundance.

(C) *Lactobacillus rhamnosus* proportional abundance.

(D) *Lactobacillus acidophilus* proportional abundance.

(E-H) Correlation between *Lactobacillus acidophilus* abundance and days after ceasing receiving supplementation.

(I) *Bifidobacterium* and *Lactobacillus* species abundance in Bif/Lacto infant samples arranged in chronological order by date of sample collection.

Individual points show each infant sample, the diamond indicates the group mean, box plots show median and interquartile ranges. Asterisks represent *p* values: *P < 0.05, **P < 0.01, ***P < 0.001

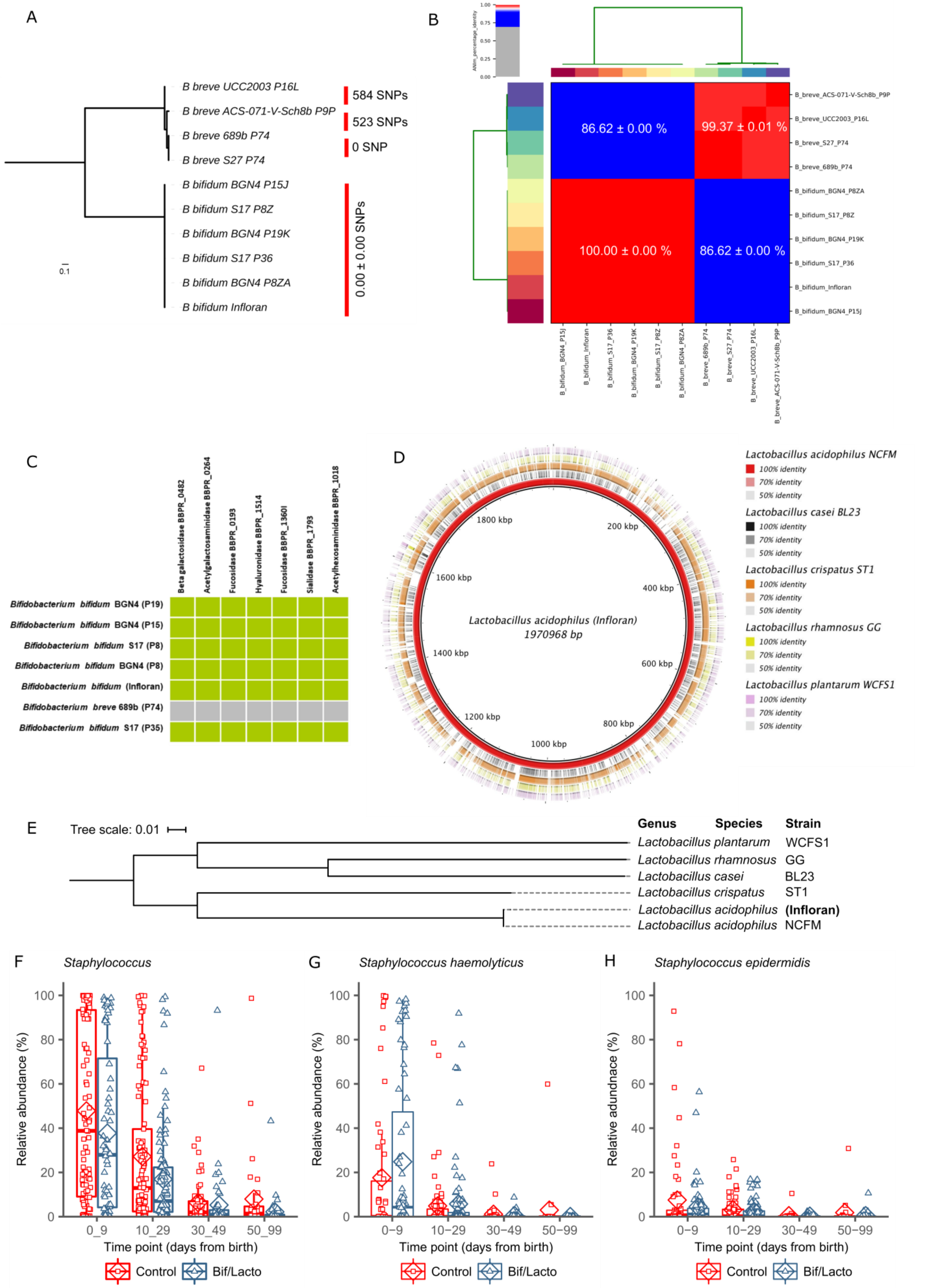
A
B breve UCC2003 P16L
B breve ACS-071-V-Sch8b P9P
B breve 689b P74
B breve S27 P74
B bifidum BGN4 P15J
B bifidum S17 P8Z
B bifidum BGN4 P19K
B bifidum S17 P36
B bifidum BGN4 P8ZA
B bifidum Infloran
584 SNPs
523 SNPs
0 SNP
0.00 ± 0.00 SNPs
0.1
B
ANIm_percentage_identity
86.62 ± 0.00 %
99.37 ± 0.01 %
100.00 ± 0.00 %
86.62 ± 0.00 %
B_breve_ACS-071-V-Sch8b_P9P
B_breve_UCC2003_P16L
B_breve_S27_P74
B_breve_689b_P74
B_bifidum_BGN4_P8ZA
B_bifidum_S17_P8Z
B_bifidum_BGN4_P19K
B_bifidum_S17_P36
B_bifidum_Infloran
B_bifidum_BGN4_P15J
C
Beta galactosidase BBPR_0482
Acetylgalactosaminidase BBPR_0264
Fucosidase BBPR_0193
Hyaluronidase BBPR_1514
Fucosidase BBPR_1360l
Sialidase BBPR_1793
Acetylhexosaminidase BBPR_1018
Bifidobacterium bifidum BGN4 (P19)
Bifidobacterium bifidum BGN4 (P15)
Bifidobacterium bifidum S17 (P8)
Bifidobacterium bifidum BGN4 (P8)
Bifidobacterium bifidum (Infloran)
Bifidobacterium breve 689b (P74)
Bifidobacterium bifidum S17 (P35)
D
Lactobacillus acidophilus (Infloran)
1970968 bp
200 kbp
400 kbp
600 kbp
800 kbp
1000 kbp
1200 kbp
1400 kbp
1600 kbp
1800 kbp
Lactobacillus acidophilus NCFM
Lactobacillus casei BL23
Lactobacillus crispatus ST1
Lactobacillus rhamnosus GG
Lactobacillus plantarum WCFS1
100% identity
70% identity
50% identity
E
Tree scale: 0.01
Genus
Species
Strain
Lactobacillus plantarum WCFS1
Lactobacillus rhamnosus GG
Lactobacillus casei BL23
Lactobacillus crispatus ST1
Lactobacillus acidophilus (Infloran)
Lactobacillus acidophilus NCFM
F
Staphylococcus
G
Staphylococcus haemolyticus
H
Staphylococcus epidermidis
Relative abundance (%)
Relative adundnace (%)
0_9
10_29
30_49
50_99
0–9
10–29
30–49
50–99
Time point (days from birth)
Control
Bif/Lacto

Figure S5 related to Figure 4. *Lactobacillus* genome comparison and *Staphylococcus* species.

(A) Mid-point rooted maximum-likelihood tree based on 6,202 SNPs in 87 core genes from 10 *Bifidobacterium* genomes.

(B) Average Nucleotide Identity pairwise comparison between 10 *Bifidobacterium* strains. Data: mean ± S.D.

(C) Heat map representing *B. bifidum* genes involved in mucin degradation.

(D) Circular genome diagrams from *Lactobacillus acidophilus* present in the oral supplementation and a subset of five *Lactobacillus* species from NCBI database. Similarity was calculated using BLAST.

(E) Core genome tree comparison from *Lactobacillus* present in the oral supplementation and five other *Lactobacillus* species from NCBI database which were found most abundant in the 16S rRNA gene data. Roary core gene alignment output was used to create a maximum likelihood (ML) phylogenetic tree.

(F) *Staphylococcus* genus relative abundance.

(G) *Staphylococcus haemolyticus* relative abundance.

(H) *Staphylococcus epidermis* relative abundance.

A

Timepoint	n	Q2Y	p
1	38	0.562(1)	0.01
2	44	0.652(0)	0.01
3	44	0.520(1)	0.01
4	24	0.465(0)	0.01
Combined	157	0.587(1)	0.01

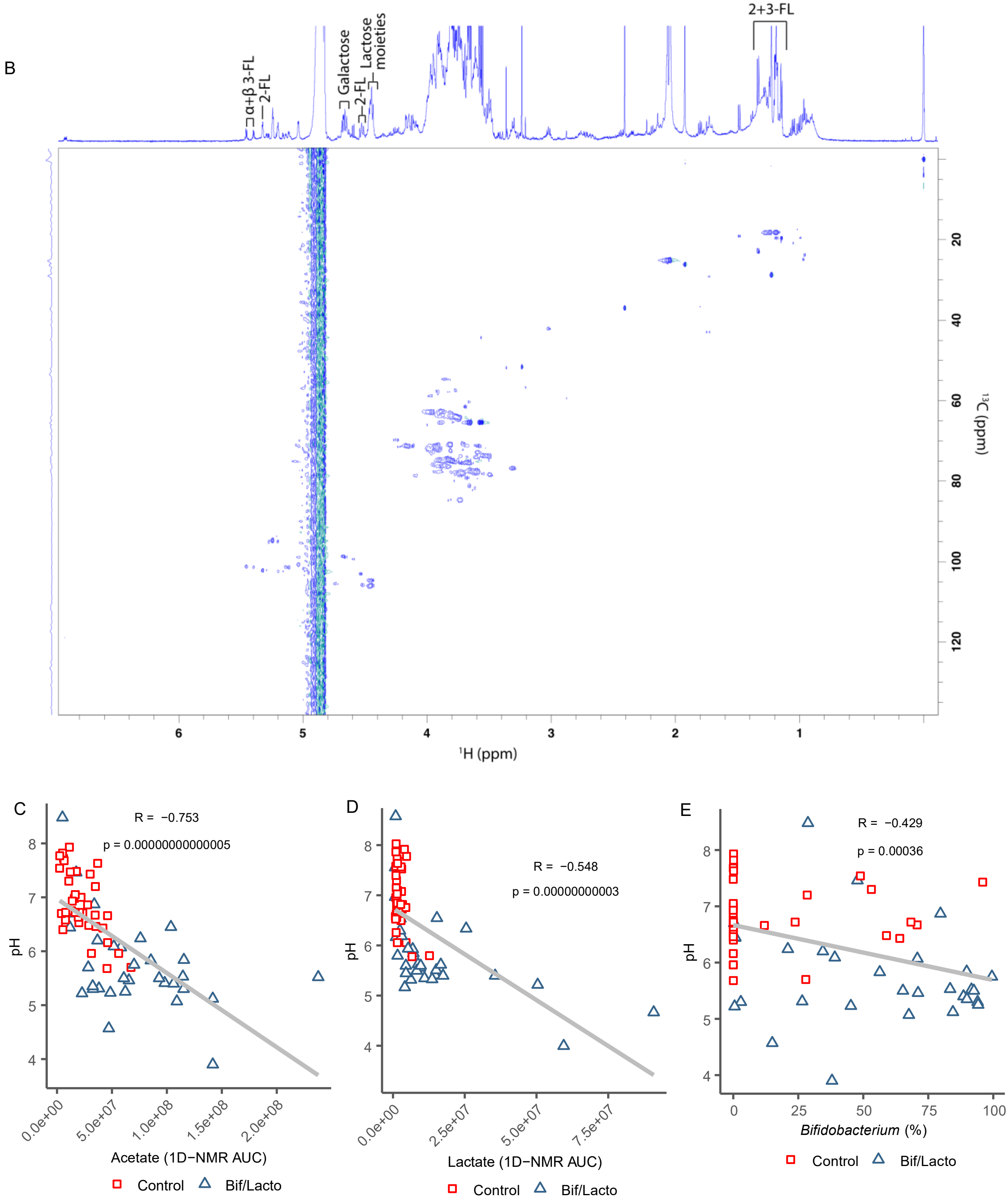

Figure S6 related to Figure 5. OPLS-DA model comparing the fecal ^1H NMR spectra of the Bif/Lacto Group and Control Group, and 2D-NMR analysis

(A) Predictive performance (Q2Y) and *p* values of the Orthogonal Projections to Latent Structures Discriminant Analysis (OPLS-DA) models comparing the Bif/Lacto and Control fecal profiles at individual time points and all timepoints combined.

(B) HSQC 2D-NMR spectrum from a study fecal sample.

(C) Spearman correlation between fecal acetate and fecal pH.

(D) Correlation between fecal lactate and fecal pH.

(E) Correlation between percentage of *Bifidobacterium* and fecal pH.

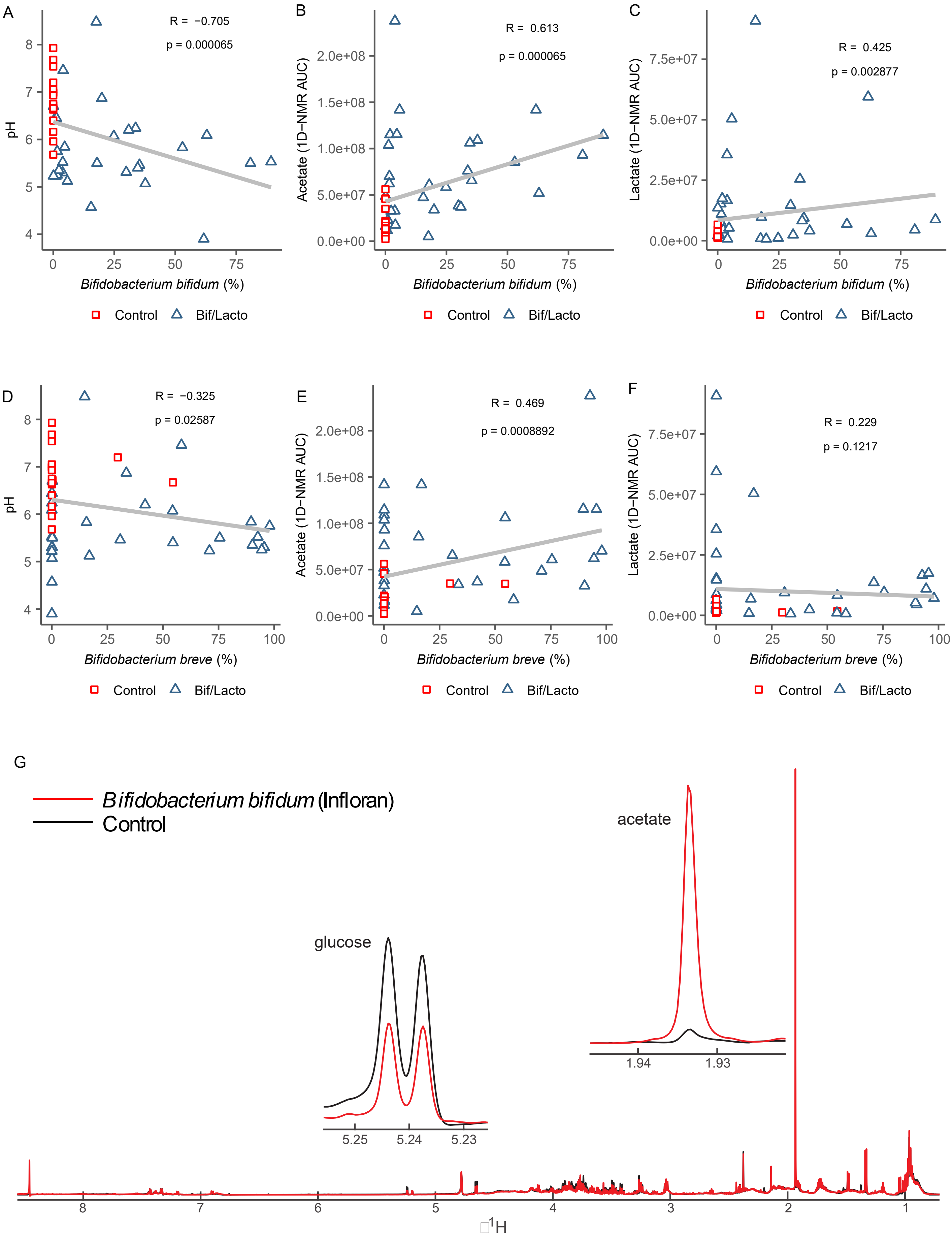
A
R = −0.705
p = 0.000065
pH
Bifidobacterium bifidum (%)
Control
Bif/Lacto
B
R = 0.613
p = 0.000065
Acetate (1D−NMR AUC)
Bifidobacterium bifidum (%)
Control
Bif/Lacto
C
R = 0.425
p = 0.002877
Lactate (1D−NMR AUC)
Bifidobacterium bifidum (%)
Control
Bif/Lacto
D
R = −0.325
p = 0.02587
pH
Bifidobacterium breve (%)
Control
Bif/Lacto
E
R = 0.469
p = 0.0008892
Acetate (1D−NMR AUC)
Bifidobacterium breve (%)
Control
Bif/Lacto
F
R = 0.229
p = 0.1217
Lactate (1D−NMR AUC)
Bifidobacterium breve (%)
Control
Bif/Lacto
G
Bifidobacterium bifidum (Infloran)
Control
acetate
glucose
1H

Figure S7 related to Figure 5. ^{1}H-NMR AUC acetate levels found in Bif/Lacto Group and Control Group.

(A) Correlation between relative abundance of *Bifidobacterium bifidum* and fecal pH.

(B) Correlation between relative abundance of *B. bifidum* and fecal acetate.

(C) Correlation between relative abundance of *B. bifidum* and fecal lactate.

(D) Correlation between relative abundance of *Bifidobacterium breve* and fecal pH.

(E) Correlation between relative abundance of *B. breve* and fecal acetate.

(F) Correlation between relative abundance of *B. breve* and fecal lactate.

(G) ^{1}H NMR spectra of the culture media from *Bifidobacterium bifidum* Infloran (red) and negative control (black).

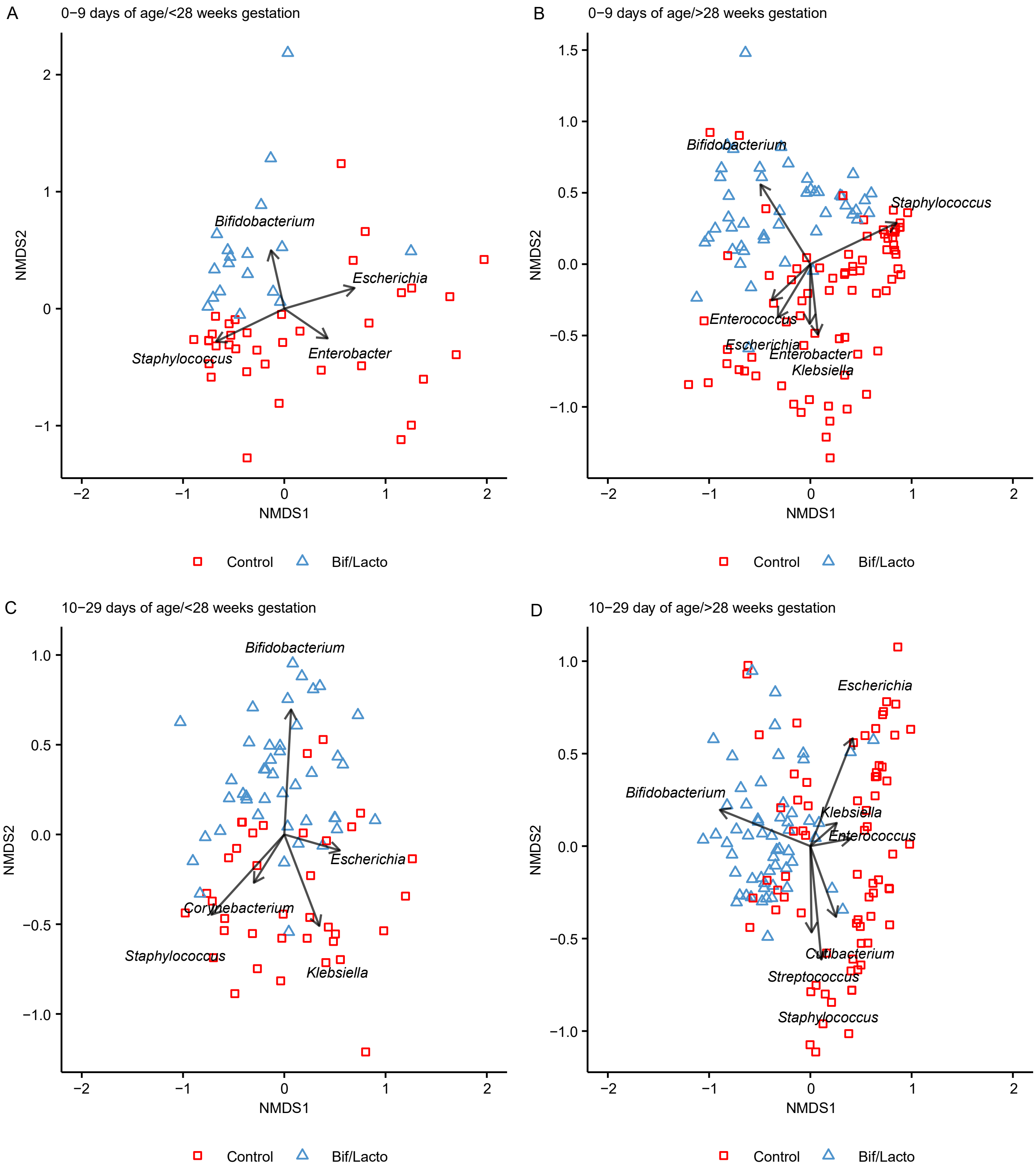

A
0−9 days of age/<28 weeks gestation
Bifidobacterium
Escherichia
Enterobacter
Staphylococcus
NMDS1
NMDS2
Control
Bif/Lacto
B
0−9 days of age/>28 weeks gestation
Bifidobacterium
Staphylococcus
Enterococcus
Escherichia
Enterobacter
Klebsiella
C
10−29 days of age/<28 weeks gestation
Bifidobacterium
Escherichia
Corynebacterium
Staphylococcus
Klebsiella
D
10−29 day of age/>28 weeks gestation
Escherichia
Bifidobacterium
Klebsiella
Enterococcus
Cutibacterium
Streptococcus
Staphylococcus

Data S1 related to Figure 1. Microbiota composition at in infants born under or at or about 28 weeks gestational age.

(A) NMDS plot at 0-9 days for infants under 28 weeks gestational age (PERMANOVA: $R^2 = 4.6$, $P = 0.03$.

(B) NMDS plot at 0-9 days for infants over 28 weeks gestational age (PERMANOVA: $R^2 = 10.6$, $P < 0.001$.

(C) NMDS plot at 10-29 days for infants under 28 weeks gestational age (PERMANOVA: $R^2 = 9.7$, $P < 0.001$.

(D) NMDS plot at 10-29 days for infants over 28 weeks gestational age (PERMANOVA: $R^2 = 16.6$, $P < 0.001$).

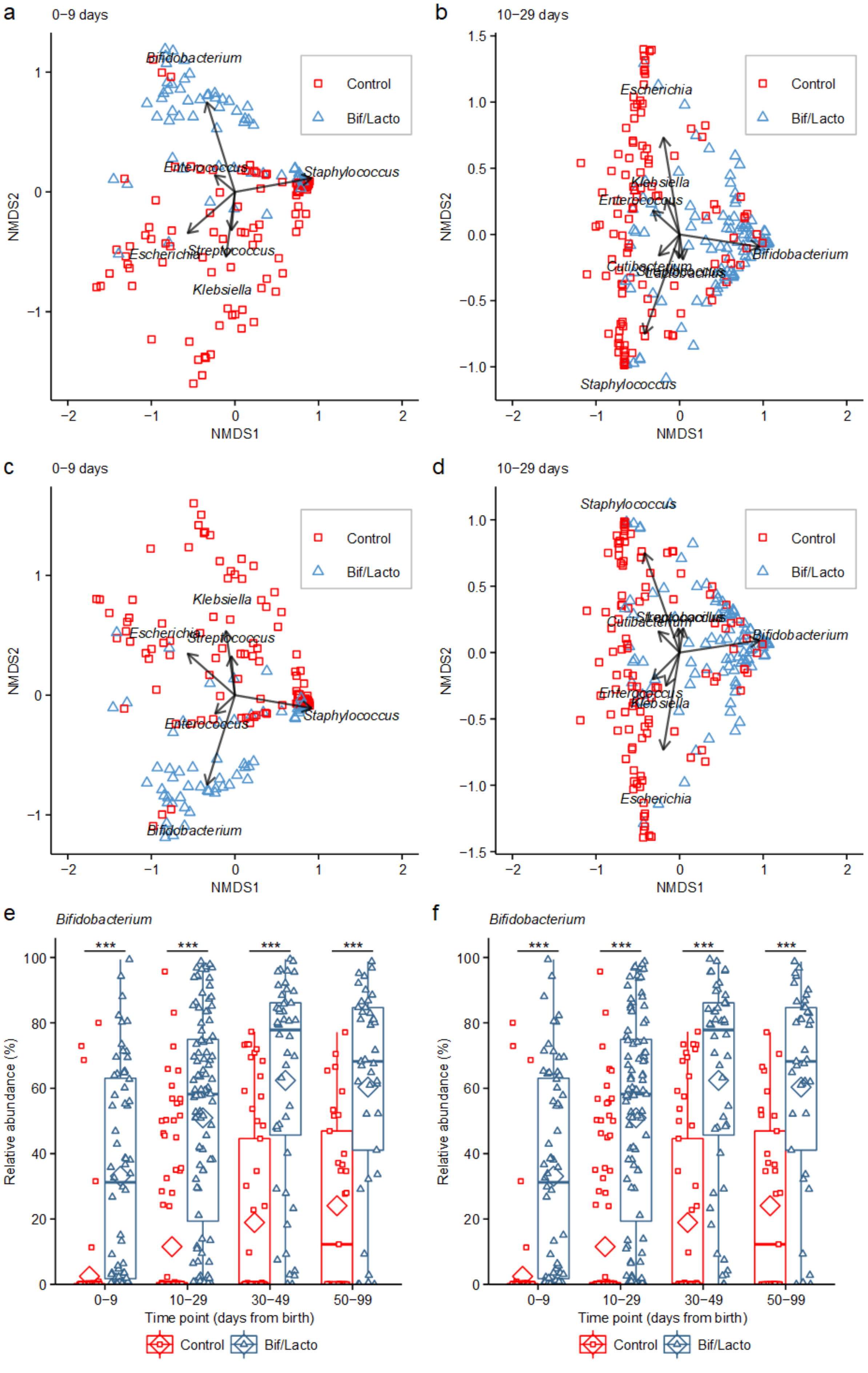

a
0–9 days
Bifidobacterium
Enterococcus
Staphylococcus
Escherichia
Streptococcus
Klebsiella
Control
Bif/Lacto
NMDS1
NMDS2
b
10–29 days
Escherichia
Klebsiella
Enterococcus
Bifidobacterium
Cutibacterium
Staphylococcus
Control
Bif/Lacto
NMDS1
NMDS2
c
0–9 days
Klebsiella
Escherichia
Streptococcus
Staphylococcus
Enterococcus
Bifidobacterium
Control
Bif/Lacto
NMDS1
NMDS2
d
10–29 days
Staphylococcus
Cutibacterium
Bifidobacterium
Enterococcus
Klebsiella
Escherichia
Control
Bif/Lacto
NMDS1
NMDS2
e
Bifidobacterium
***
Relative abundance (%)
0–9
10–29
30–49
50–99
Time point (days from birth)
Control
Bif/Lacto
f
Bifidobacterium
***
Relative abundance (%)
0–9
10–29
30–49
50–99
Time point (days from birth)
Control
Bif/Lacto

Data S2 related to Figure 1. NMDS plots and Bifidobacterium relative abundance using data normalised with either mean log-transformation or variance-stabilizing transformation.

(A) NMDS plot of fecal microbiota at 0-9 days with sequence data normalised using mean log-normalised transformation.

(B) NMDS plot of fecal microbiota at 10-29 days with sequence data normalised using mean log-normalised transformation.

(C) NMDS plot of fecal microbiota at 0-9 days with sequence data normalised using variance-stabilizing transformation.

(D) NMDS plot of fecal microbiota at 10-29 days with sequence data normalised using variance-stabilizing transformation.

(E) Bifidobacterium relative abundance with sequence data normalised using mean log-normalised transformation

(F) Bifidobacterium relative abundance with sequence data normalised using variance-stabilizing transformation.

Asterisks represent P values: *P < 0.05, **P < 0.01, ***P < 0.001.

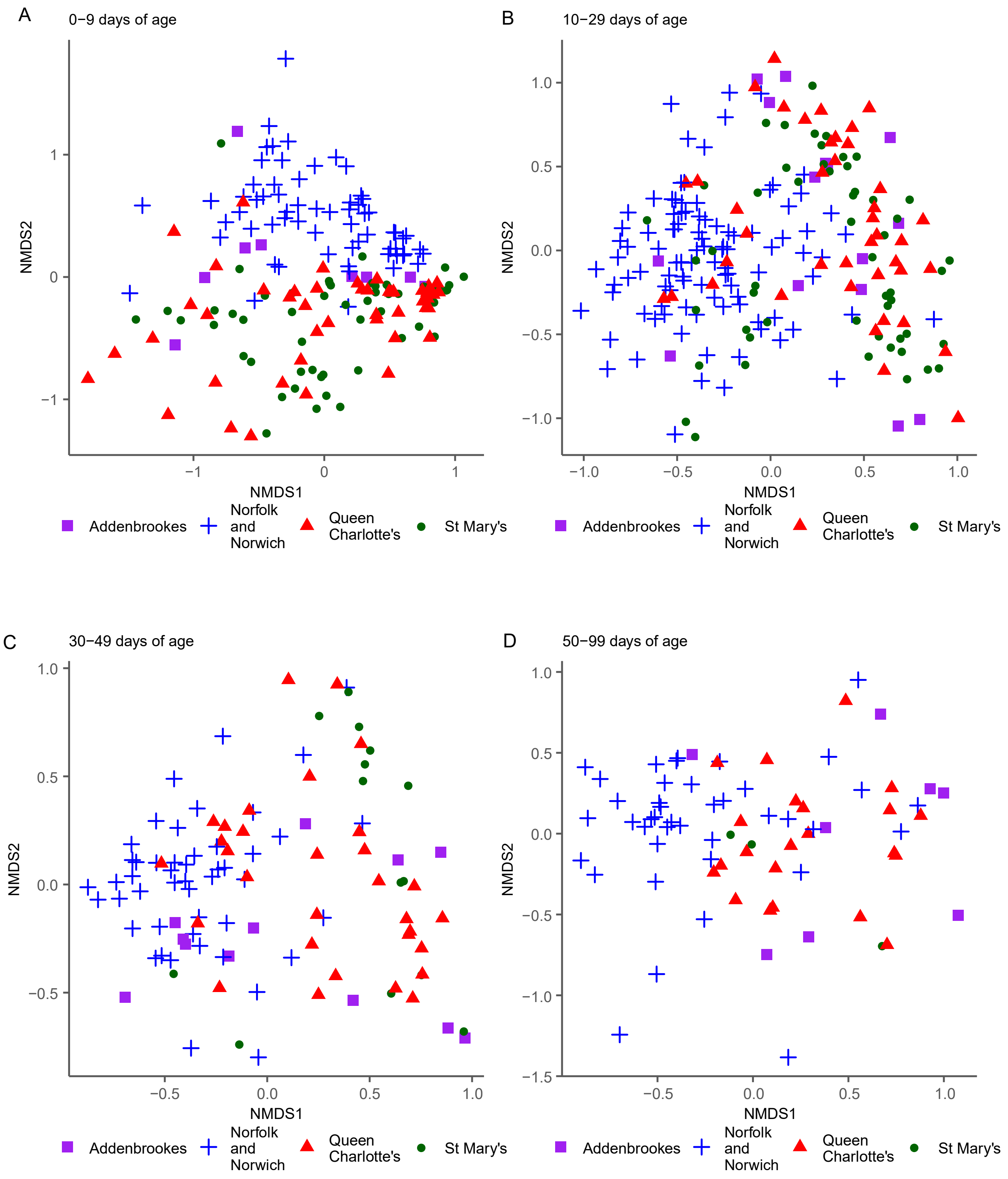
A
0−9 days of age
B
10−29 days of age
C
30−49 days of age
D
50−99 days of age
NMDS1
NMDS2
Addenbrookes
Norfolk and Norwich
Queen Charlotte's
St Mary's

Data S3 related to Figure 1. Non-metric multidimensional scaling (NMDS) comparing microbiota composition between individual Bif/Lacto (Norfolk and Norwich) and Control hospital NICUs.

(A) NMDS infant fecal microbiota composition compared by NICU at 0-9 days.

(B) NMDS infant fecal microbiota composition compared by NICU at 10-29 days.

(C) NMDS infant fecal microbiota composition compared by NICU at 30-49 days.

(D) NMDS infant fecal microbiota composition compared by NICU at 50-99 days.

NMDS analysis clustered using Bray-Curtis dissimilarity.

Bif/Lacto group: Norfolk and Norwich. Control group: St Mary's, Queen Charlotte's, and Addenbrookes.

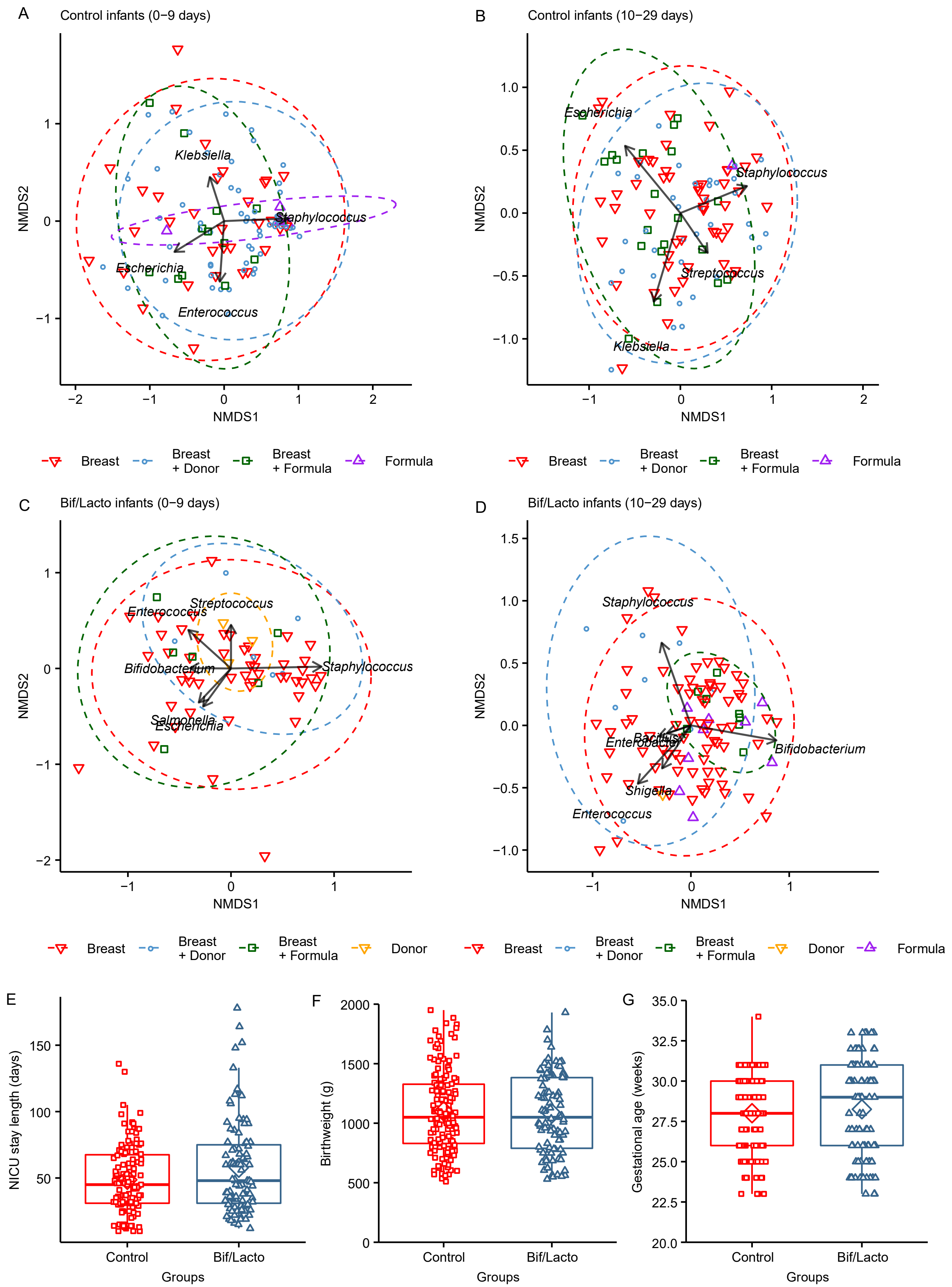

A
Control infants (0–9 days)
Klebsiella
Staphylococcus
Escherichia
Enterococcus
NMDS1
NMDS2
B
Control infants (10–29 days)
Escherichia
Staphylococcus
Streptococcus
Klebsiella
Breast
Breast + Donor
Breast + Formula
Formula
C
Bif/Lacto infants (0–9 days)
Enterococcus
Streptococcus
Bifidobacterium
Staphylococcus
Salmonella
Escherichia
D
Bif/Lacto infants (10–29 days)
Staphylococcus
Bacillus
Enterobacter
Bifidobacterium
Shigella
Enterococcus
Donor
E
NICU stay length (days)
F
Birthweight (g)
G
Gestational age (weeks)
Control
Bif/Lacto
Groups

Data S4 related to Figure 2. Effects of birthweight, antibiotics, delivery mode and diet on Bifidobacterium.

(A) Control infant fecal microbiota composition compared by diet at 0-9 days.

(B) Control infant fecal microbiota composition compared by diet at 10-29 days.

(C) Bif/Lacto infant fecal microbiota composition compared by diet at 0-9 days.

(D) Bif/Lacto infant fecal microbiota composition compared by NICU at 10-29 days.

NMDS analysis clustered using Bray-Curtis dissimilarity. Ellipses show 95% confidence interval for each group.

(E) Length of infant stay in NICU.

(F) Infant birth weight.

(G) Infant gestational age at birth.

Box plots show mean (diamond) and median (solid line) with point showing individual infants.

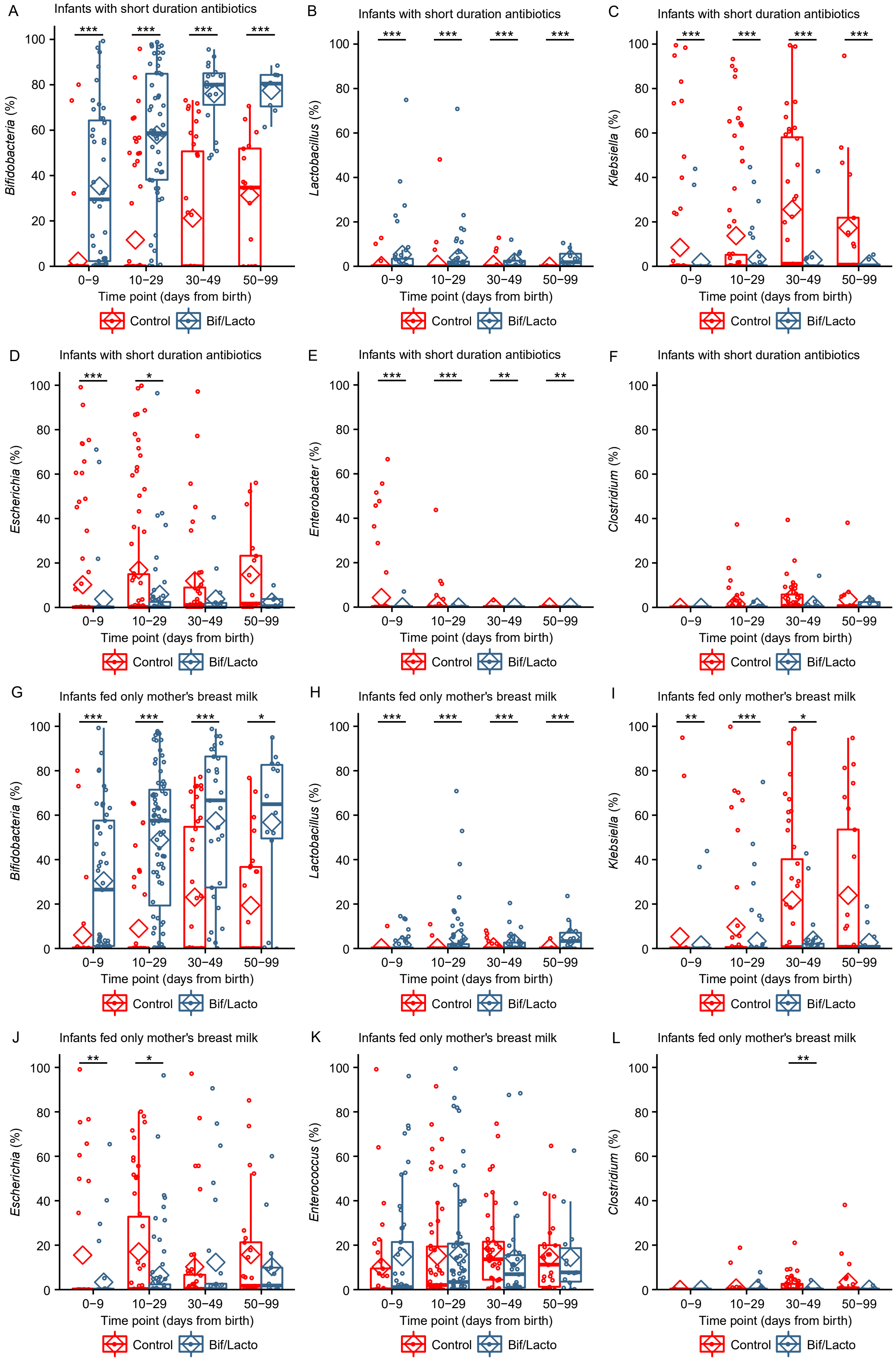

A Infants with short duration antibiotics
B Infants with short duration antibiotics
C Infants with short duration antibiotics
D Infants with short duration antibiotics
E Infants with short duration antibiotics
F Infants with short duration antibiotics
G Infants fed only mother's breast milk
H Infants fed only mother's breast milk
I Infants fed only mother's breast milk
J Infants fed only mother's breast milk
K Infants fed only mother's breast milk
L Infants fed only mother's breast milk
Bifidobacteria (%)
Lactobacillus (%)
Klebsiella (%)
Escherichia (%)
Enterobacter (%)
Clostridium (%)
Enterococcus (%)
0-9
10-29
30-49
50-99
Time point (days from birth)
Control
Bif/Lacto

Data S5 related to Figure 2. Sensitivity analysis of genus abundance restricted to only infants that received short duration antibiotic treatment or to infants that were only fed mothers' breast milk at time of sample collection.

(A) Relative abundance of *Bifidobacterium.*

(B) Relative abundance of *Lactobacillus.*

(C) Relative abundance of *Klebsiella.*

(D) Relative abundance of *Escherichia.*

(E) Relative abundance of *Enterobacter.*

(F) Relative abundance of *Clostridium*.

(G) Relative abundance of *Bifidobacterium.*

(H) Relative abundance of *Lactobacillus.*

(I) Relative abundance of *Klebsiella.*

(J) Relative abundance of *Escherichia.*

(K) Relative abundance of *Enterobacter.*

(L) Relative abundance of *Clostridium*.

Individual points highlight individual infant samples, diamonds indicate the group mean, box plots show group median and interquartile range. Asterisks represent p values: * = $P < 0.05$, ** = $P < 0.01$ *** = $P < 0.001$.

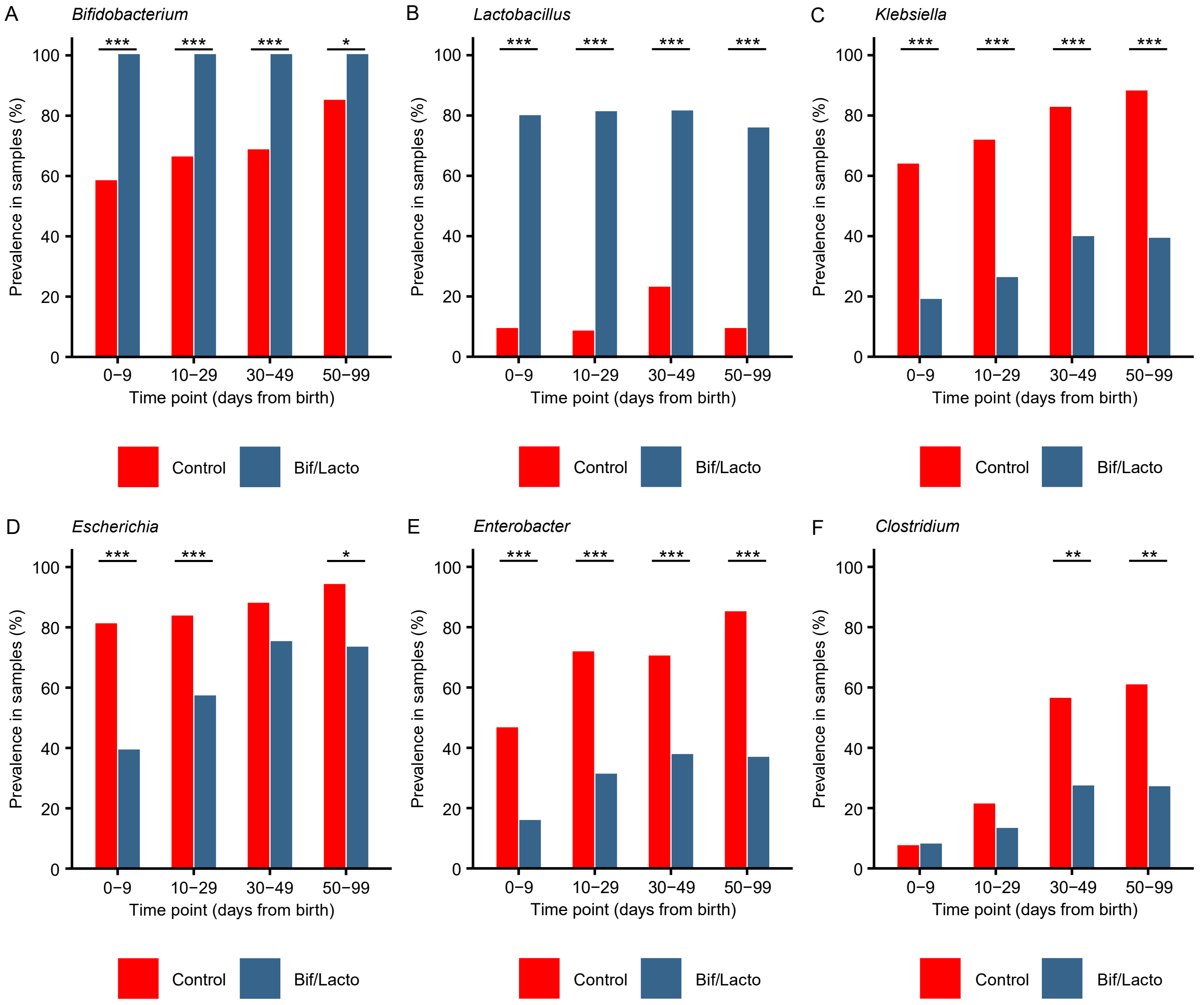

Data S6 related to Figure 2. Prevalence of genus in infants.

(A) Prevalence of *Bifidobacterium.*

(B) Prevalence of *Lactobacillus.*

(C) Prevalence of *Klebsiella.*

(D) Prevalence of *Escherichia .*

(E) Prevalence of *Enterobacter.*

(F) Prevalence of *Clostridium*.

Prevalence of a genus counted as one or more sequence read detected and absence counted as zero sequence read detected in each sample.

Significance tested using Fisher's Exact Test. Asterisks represent *p* values: * = P < 0.05, ** = P < 0.01 *** = P < 0.001.

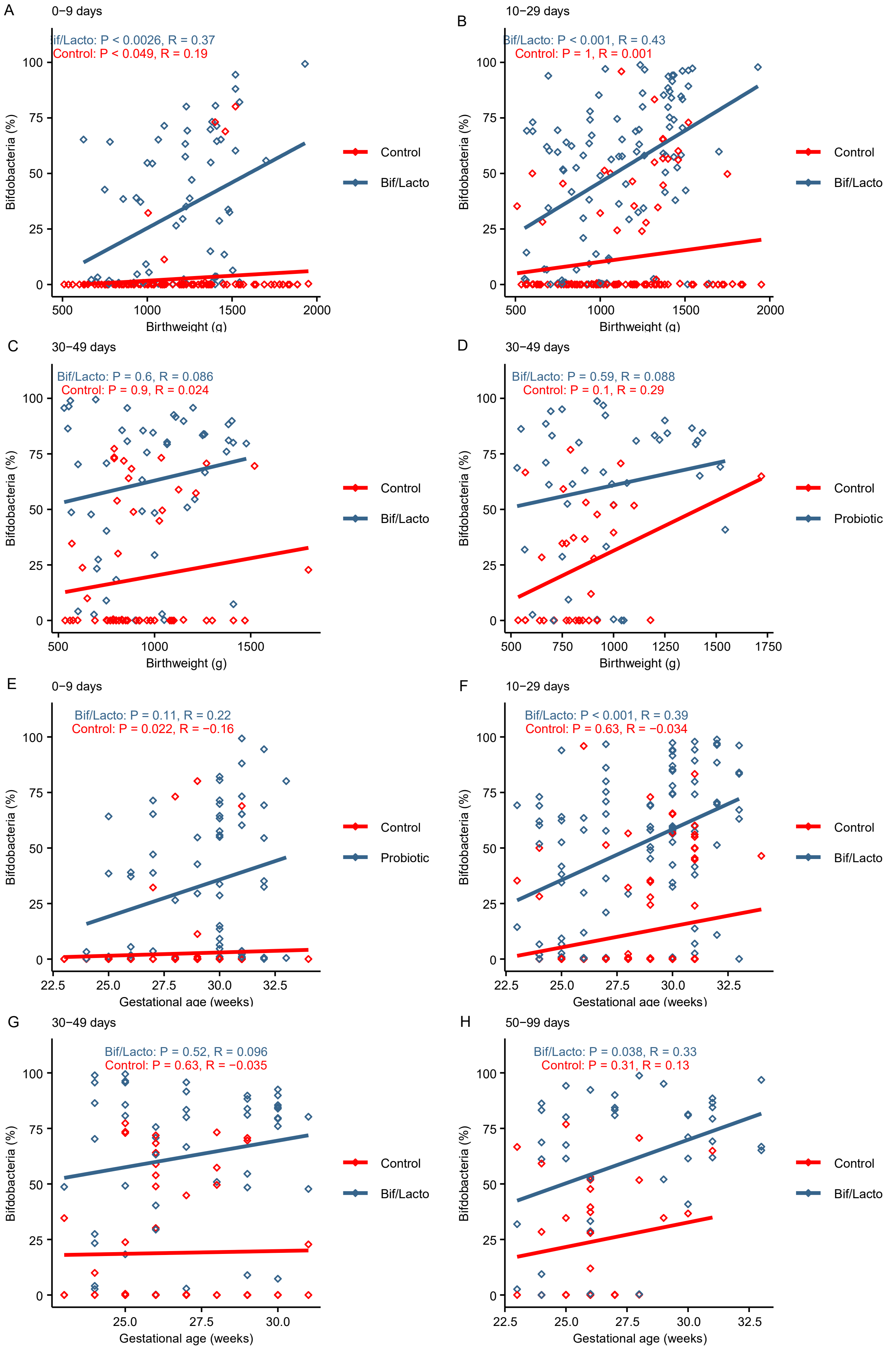
A
0−9 days
Bif/Lacto: P < 0.0026, R = 0.37
Control: P < 0.049, R = 0.19
Bifdobacteria (%)
Birthweight (g)
Control
Bif/Lacto
B
10−29 days
Bif/Lacto: P < 0.001, R = 0.43
Control: P = 1, R = 0.001
Bifdobacteria (%)
Birthweight (g)
Control
Bif/Lacto
C
30−49 days
Bif/Lacto: P = 0.6, R = 0.086
Control: P = 0.9, R = 0.024
Bifdobacteria (%)
Birthweight (g)
Control
Bif/Lacto
D
30−49 days
Bif/Lacto: P = 0.59, R = 0.088
Control: P = 0.1, R = 0.29
Bifdobacteria (%)
Birthweight (g)
Control
Probiotic
E
0−9 days
Bif/Lacto: P = 0.11, R = 0.22
Control: P = 0.022, R = −0.16
Bifdobacteria (%)
Gestational age (weeks)
Control
Probiotic
F
10−29 days
Bif/Lacto: P < 0.001, R = 0.39
Control: P = 0.63, R = −0.034
Bifdobacteria (%)
Gestational age (weeks)
Control
Bif/Lacto
G
30−49 days
Bif/Lacto: P = 0.52, R = 0.096
Control: P = 0.63, R = −0.035
Bifdobacteria (%)
Gestational age (weeks)
Control
Bif/Lacto
H
50−99 days
Bif/Lacto: P = 0.038, R = 0.33
Control: P = 0.31, R = 0.13
Bifdobacteria (%)
Gestational age (weeks)
Control
Bif/Lacto

Data S7 related to Figure 3. *Bifidobacterium* relative abundance plotted against birthweight or gestational age.

(A) *Bifidobacterium* relative abundance plotted against birth weight at 0-9 days.

(B) *Bifidobacterium* relative abundance plotted against birth weight at 10-29 days.

(C) *Bifidobacterium* relative abundance plotted against birth weight at 30-49 days.

(D) *Bifidobacterium* relative abundance plotted against birth weight at 40-99 days.

(E) *Bifidobacterium* relative abundance plotted against gestational age at 0-9 days.

(F) *Bifidobacterium* relative abundance plotted against gestational age at 10-29 days.

(G) *Bifidobacterium* relative abundance plotted against gestational age at 30-49 days.

(H) *Bifidobacterium* relative abundance plotted against gestational age at 40-99 days.
